# Repair of Oxidative DNA Damage, Cell-Cycle Regulation and Neuronal Death May Influence the Clinical Manifestation of Alzheimer’s Disease

**DOI:** 10.1371/journal.pone.0099897

**Published:** 2014-06-17

**Authors:** Aderbal R. T. Silva, Ana Cecília Feio Santos, Jose M. Farfel, Lea T. Grinberg, Renata E. L. Ferretti, Antonio Hugo Jose Froes Marques Campos, Isabela Werneck Cunha, Maria Dirlei Begnami, Rafael M. Rocha, Dirce M. Carraro, Carlos Alberto de Bragança Pereira, Wilson Jacob-Filho, Helena Brentani

**Affiliations:** 1 Laboratory of Clinical Pathology - Laboratory of Medical Investigations 23 (LIM 23), Department and Institute of Psychiatry, University of São Paulo, Medical School, São Paulo, Brazil; 2 Brazilian Brain Bank of the Aging Brain Study Group - Laboratory of Medical Investigations 22 (LIM 22), University of São Paulo, Medical School, São Paulo, Brazil; 3 Division of Geriatrics, University of São Paulo, Medical School, São Paulo, Brazil; 4 Memory and Aging Center, Department of Neurology, University of California San Francisco, San Francisco, California, United States of America; 5 Department of Pathology, A. C. Camargo Cancer Center, São Paulo, Brazil; 6 Research Center (CIPE), A. C. Camargo Cancer Center, São Paulo, Brazil; 7 Mathematics and Statistics Institute, University of São Paulo, São Paulo, Brazil; Boston University School of Medicine, United States of America

## Abstract

Alzheimer’s disease (AD) is characterized by progressive cognitive decline associated with a featured neuropathology (neuritic plaques and neurofibrillary tangles). Several studies have implicated oxidative damage to DNA, DNA repair, and altered cell-cycle regulation in addition to cell death in AD post-mitotic neurons. However, there is a lack of studies that systematically assess those biological processes in patients with AD neuropathology but with no evidence of cognitive impairment. We evaluated markers of oxidative DNA damage (8-OHdG, H2AX), DNA repair (p53, BRCA1, PTEN), and cell-cycle (Cdk1, Cdk4, Cdk5, Cyclin B1, Cyclin D1, p27^Kip1^, phospho-Rb and E2F1) through immunohistochemistry and cell death through TUNEL in autopsy hippocampal tissue samples arrayed in a tissue microarray (TMA) composed of three groups: I) “*clinical-pathological* AD” (CP-AD) - subjects with neuropathological AD (Braak≥IV and CERAD = B or C) and clinical dementia (CDR≥2, IQCODE>3.8); II) “*pathological* AD” (P-AD) - subjects with neuropathological AD (Braak≥IV and CERAD = B or C) and without cognitive impairment (CDR 0, IQCODE<3.2); and III) “normal aging” (N) - subjects without neuropathological AD (Braak≤II and CERAD 0 or A) and with normal cognitive function (CDR 0, IQCODE<3.2). Our results show that high levels of oxidative DNA damage are present in all groups. However, significant reductions in DNA repair and cell-cycle inhibition markers and increases in cell-cycle progression and cell death markers in subjects with CP-AD were detected when compared to both P-AD and N groups, whereas there were no significant differences in the studied markers between P-AD individuals and N subjects. This study indicates that, even in the setting of pathological AD, healthy cognition may be associated with a preserved repair to DNA damage, cell-cycle regulation, and cell death in post-mitotic neurons.

## Introduction

Alzheimer’s disease (AD) is the most frequent cause of dementia in the elderly, accounting for approximately 70% of dementia cases [1], [2]. AD is clinically characterized by a progressive decline of cognitive function, personality changes, and psychiatric symptoms, evolving through many years until it culminates, in advanced phases, with a loss of autonomy, functional dependency for activities of daily life and finally death. The brains of patients with AD, in addition to showing synaptic loss, are histopathologically characterized by two hallmark lesions – plaques containing amyloid-β peptides (Aβ) and neurofibrillary tangles composed of hyperphosphorylated forms of the microtubule-associated protein tau [3].

Although there is evidence showing that the majority of persons identified as having clinical AD meet the neuropathological criteria for AD [4]–[8], the correlation between those neuropathological lesions and cognition is relatively poor. Among individuals who showed no overt evidence of cognitive impairment in the final assessment prior to death, post-mortem studies showed a significant proportion of subjects with neuropathological diagnoses of AD [9], [10]. Molecular neuroimaging and CSF biomarker studies have demonstrated that 20–40% of older subjects with no cognitive impairment display a significant accumulation of Aβ in the brain [11]–[15]. In addition, more recent large-scale epidemiological studies have shown discordance of cognition with AD pathology [16]–[20]. Therefore, it is evident that healthy cognition can occur even with significant accumulations of pathological features.

Although the neurobiological basis of such discordance between pathology and clinical outcome is not yet understood, there may be compensatory mechanisms that protect such individuals against the clinical emergence of AD because they carry a high burden of histopathological lesions but can support them without cognitive decline, remaining resilient [21]–[23]. To uncover the molecular aspects that underlie these pathophysiological processes and resilient brain aging, we performed a gene expression study among subjects with 1) clinical dementia and AD pathology (CP-AD), 2) AD pathology and healthy cognition (P-AD), and normal individuals (N) [24]. Genes involved in oxidative stress and in DNA damage/repair were found to be coupled with AD neuropathology. Moreover, through gene classifiers, we revealed that genes related to the cell-cycle were able to discriminate between CP-AD and P-AD subjects, indicating a possible role for those biological processes in brain resilience.

DNA injury due to oxidative stress has been confirmed in various studies to have a role in the pathogenesis of AD [25]. Different biomarkers of oxidative damage to DNA have been assessed in AD brains, but the most popular for assays is the base 8-hydroxy-2-deoxyguanosine, which is increased in AD brain samples compared with age-matched controls [26], [27]. Consequently, these results have led to intensive research on alterations in DNA repair proteins involved in the repair of such lesions in patients with AD, including p53, PTEN and BRCA1 [28]–[33]. Furthermore, changes in the expression of cell-cycle proteins (cyclins D [34] and B [34]–[36], Cdk4 [37], Cdk1 [38], p27 [39], E2F1 [40], and phosphorylated retinoblastoma protein [41]) have been noted in neurons of post-mortem brain specimens from persons with AD. Of note, these proteins are unusual in postmitotic cells. Another protein (Cdk5), which is not associated with the cell-cycle, has been suggested as a cell-cycle suppressor in post-mitotic neurons [42], [43], with ectopic subcellular redistribution in neurons of cases affected with AD [44], [45]. In addition, cell-cycle studies have shown that ectopic expression of cell-cycle markers is associated with neuronal cell death [46]–[48].

It is important to note that although oxidative damage to DNA (ODD), DNA repair (DR), cell-cycle (CC) and cell death (CD) have been widely explored in individuals with AD (with both AD pathology and clinical dementia, CP-AD), there has been little postmortem investigation of neurobiological processes in individuals with normal cognition despite AD pathology (P-AD). Therefore, our aim in this study was to verify whether there are differences among CP-AD, P-AD, and normal individuals (no AD pathology and no clinical dementia) related to ODD, DR, CC, and CD. We observed reduced levels of DNA repair and cell-cycle inhibition markers, as well as elevated levels of cell-cycle progression markers in association with increased levels of cell death in post-mitotic neurons of clinical and pathological AD individuals; in contrast, individuals with AD neuropathology but no evidence of cognitive impairment (P-AD) present a similar expression profile to individuals with normal aging.

## Materials and Methods

### Tissue Samples and Neuropathological/Cognitive Assessment

Hippocampal tissue samples were obtained post-mortem from the Brain Bank of the Brazilian Aging Brain Study Group [49]. After death, a trained gerontologist interviewed a knowledgeable informant who had at least weekly contact with the deceased subjects. Past medical history, cognitive performance and functional status were determined for each subject [50]. Cognition was assessed with the application of both the Clinical Dementia Rating scale (CDR) [51] and the Informant Questionnaire of the Cognitive Decline on the Elderly (IQCODE) [52], [53]. The CDR was applied as a semi-structured questionnaire, and this method of application, exclusively with the informant, has been evaluated and published [54]. All informants voluntarily signed an Informed Consent Form and consented to provide all clinical information requested.

Human autopsy brain tissue was fixed in 4% paraformaldehyde and used for neuropathological evaluation. Neuropathological examinations were performed using immunohistochemistry, according to internationally accepted criteria [49]. Neurofibrillary tangles (NFTs) and neuritic plaques (NPs) were assessed by a skilled neuropathologist in accordance with the Braak stage system [55] and the Consortium to Establish a Registry for Alzheimer’s Disease (CERAD) [56], respectively. Cases with a Braak stage≥IV or the presence of moderate or frequent neuritic plaques in one or more neocortical regions (CERAD = B or C) were classified as meeting criteria for AD. The neuropathologist was blinded to all clinical information.

Based on pathological and clinical criteria, subjects were categorized into three groups: I) 19 subjects with neuropathological AD (Braak≥IV and CERAD = B or C) and clinical dementia (CDR≥2, IQCODE>3.8), termed “*clinical-pathological* AD” (CP*-*AD); II) 12 subjects with neuropathological AD (Braak≥IV and CERAD = B or C) and without cognitive impairment (CDR = 0, IQCODE<3.2), termed “*pathological* AD” (P-AD); and III) 31 subjects without neuropathological AD (Braak≤II and CERAD 0 or A) and with normal cognitive function (CDR = 0, IQCODE<3.2), termed “normal aging individuals” (N). The neuropathological and clinical data and post-mortem interval of each case can be visualized in Table 1.

**Table 1:** Summary of selected cases.

Sample ID	Gender	Age	Braak	CERAD	CDR	PMI
CP-AD1	F	98	3	B	2	15.1
CP-AD2	M	92	6	C	3	14.8
CP-AD3	F	82	5	C	3	20.8
CP-AD4	F	82	6	C	3	15.3
CP-AD5	F	87	4	B	2	12.0
CP-AD6	M	86	6	B	3	17.6
CP-AD7	F	81	5	B	2	13.6
CP-AD8	F	81	5	C	2	14.0
CP-AD9	F	83	6	C	3	12.8
CP-AD10	F	90	4	B	3	11.3
CP-AD11	F	99	5	B	3	18.3
CP-AD12	F	81	6	C	3	15.9
CP-AD13	F	94	4	B	3	12.8
CP-AD14	M	84	5	C	3	12.3
CP-AD15	F	82	6	C	3	20.0
CP-AD16	F	81	5	C	3	18.5
CP-AD17	F	83	6	C	3	10.8
CP-AD18	F	82	5	C	2	11.8
CP-AD19	F	87	5	B	3	17.7
P-AD1	M	82	4	B	0	11.3
P-AD2	M	80	4	B	0	11.8
P-AD3	M	91	4	B	0	23.2
P-AD4	M	81	4	B	0	12.3
P-AD5	F	97	3	C	0	12.1
P-AD6	F	83	3	B	0	16.1
P-AD7	F	89	4	C	0	13.3
P-AD8	M	82	4	B	0	13.9
P-AD9	F	87	5	C	0	11.1
P-AD10	F	85	5	C	0	9.6
P-AD11	F	86	6	C	0	16.0
P-AD12	F	81	5	C	0	13.0
N1	M	80	3	0	0	19.7
N2	F	83	3	0	0	10.0
N3	M	86	2	0	0	15.5
N4	M	82	2	0	0	16.7
N5	M	83	2	0	0	16.1
N6	F	82	3	0	0	14.2
N7	F	83	0	0	0	16.5
N8	M	89	2	0	0	14.7
N9	M	81	2	0	0	9.0
N10	M	83	1	0	0	17.9
N11	F	82	2	A	0	11.3
N12	M	80	2	0	0	16.9
N13	F	81	3	A	0	14.1
N14	F	80	2	0	0	12.8
N15	M	95	3	0	0	15.3
N16	F	94	2	0	0	12.3
N17	M	83	1	0	0	12.5
N18	F	83	2	0	0	13.1
N19	F	83	2	A	0	21.6
N20	F	81	1	0	0	11.9
N21	F	93	2	0	0	13.7
N22	F	82	2	0	0	10.8
N23	F	82	2	0	0	15.6
N24	F	82	1	0	0	14.8
N25	F	86	2	0	0	10.7
N26	F	86	2	0	0	23.0
N27	M	89	2	0	0	14.2
N28	M	92	3	A	0	10.4
N29	M	83	0	0	0	15.4
N30	M	90	2	0	0	13.0
N31	M	83	2	0	0	13.7

Subjects were divided into three groups, based on neuropathological and clinical criteria: clinical-pathological Alzheimer’s disease (CP-AD), pathological Alzheimer’s disease (P-AD), and normal aging (N). Sample ID, sample identification; Age, age at death in years; F, female; M, male; Braak, Braak stage; CERAD, Consortium to Establish a Registry for Alzheimer’s Disease score; CDR, Clinical Dementia Ratio score; PMI, post-mortem interval in hours.

This study was approved by the Ethical Board for Research Project Analysis (CAPPesq) of the University of São Paulo Medical School (research protocol 285/04) and by the Ethical Committee for Research (CEP) of the A. C. Camargo Cancer Center (research protocol 1232/09) and was conducted in accordance with the Helsinki Declaration.

### Construction of Tissue Microarray

Using the tissue microarray (TMA) technology, hippocampal samples (CA1 region) from 62 subjects (18 CP-AD, 12 P-AD, and 31 N) were arrayed in one recipient paraffin block using a manual arraying instrument (Manual Tissue Arrayer 1, Beecher Instruments Microarray Technology, Silver Spring, MI, USA). This instrument was utilized for creating holes in the recipient array block and for acquiring tissue cores from the donor block. The donor block was manually positioned for sampling based on a visual alignment with the corresponding HE-stained section on a slide by a pathologist. The needle was used to retrieve a cylindrical sample from a selected region in the donor block and to extrude the sample core directly into the recipient block with defined array coordinates. After the block construction was completed, 4-µm sections of the resulting tissue microarray block were cut with a microtome. An adhesive tape system (Instrumedics, Hackensack, New Jersey) was used for sectioning the array block. The microtome knife cuts underneath a piece of tape that is placed over the block surface. The thin tissue section adheres to the tape, which is then rolled on an adhesive-coated microscope slide to transfer the section to the slide.

### Immunohistochemistry

TMA sections were immersed in xylene to remove the paraffin and then hydrated through graded ethanol solutions. Antigen retrieval was performed with Tris-EDTA buffer (pH 9.0) and boiled in a pressure-cooker for 4 min. Endogenous peroxidase activity was removed with 3% hydrogen peroxide (H_2_O_2_) three times for 10 min. The sections were blocked with serum-free protein block (Dako, Carpinteria, USA) at room temperature for 20 min to prevent non-specific binding and then incubated with the primary antibodies (Table 2) at room temperature for 2 h. Following incubation with secondary antibodies (Advanced™ HRP Link, DAKO®), and then with antibodies polymerized with horseradish peroxidase (Advanced™ HRP Enzyme (DAKO®), staining was performed by incubating the slides in 3,3′-diaminobenzidine tetrachloride (Dako, Carpinteria, USA). The slides were then lightly counterstained with hematoxylin, dehydrated in absolute ethanol and xylene, and then mounted with coverslips using a permanent mounting medium.

**Table 2:** Primary antibodies.

Primary Antibody	Clonality	Dilution	Source
Anti-8-OHdG	Monoclonal (N45.1)	1/600	JaICA
Anti-λ-H2AX (Ser139)	Polyclonal	1/100	Novus Biologicals
Anti-BRCA1	Monoclonal (GLK-2)	1/200	Dako
Anti-p53	Monoclonal (DO-7)	1/3000	Dako
Anti-PTEN	Monoclonal (6H2.1)	1/100	Cascade BioScience
Anti-phospho-AKT (Ser473)	Monoclonal (587F11)	1/75	Cell Signaling
Anti-Cdk1	Monoclonal (A17.1.1)	1/500	NeoMarkers
Anti-Cdk4	Polyclonal	1/400	Abcam
Anti-Cdk5	Monoclonal (DC17+DC34)	1/200	Abcam
Anti-Cyclin B1	Monoclonal (V152)	1/300	Dako
Anti-Cyclin D1	Monoclonal (SP4)	1/25	Cell Marque
Anti-E2F-1	Monoclonal (KH95)	1/200	NeoMarkers
Anti-p27^Kip1^	Monoclonal (SX53G8)	1/100	Dako
Anti-Rb	Monoclonal (13A10)	1/200	Leica BioSystems
Anti-phospho-Rb (Ser608)	Monoclonal (51B7)	1/200	Abcam

### Apoptosis Assay

Detection of cell death was performed using the TACS 2 TdT®-DAB *In Situ* Apoptosis Detection Kit (TREVIGEN®) according to the manufacturer’s instructions. This technique, based on TUNEL, detects DNA fragmentation resulting from apoptotic signaling cascades. This assay is based on the presence of DNA fragments, which can be identified with terminal deoxynucleotidyltransferase, an enzyme that catalyzes the addition of labeled dUTPs with a secondary antibody conjugated to horseradish peroxidase, generating a brown coloration when reacted with DAB.

### Data Analysis

After performing immunohistochemical reactions, slides were digitalized on a ScanScope XT device (Aperio Technologies), and then the full-slide image was segmented by Spectrum™ software (Aperio Technologies) to generate an image of each core (sample). Subsequently, these images were used to manually count neurons using the ‘*Events*’ tool of ZEN lite software (ZEISS). Identification of neurons was based on their typical morphological parameters [57]. First, to estimate the numbers of neurons in the sampled regions, pyramidal neurons from hippocampi of five control subjects (normal group) at three different cut levels were counted, as illustrated in Figure S1, and then averaged. Second, neurons with positive immunoreactivity were counted, considering the nuclear and cytoplasmic staining separately (Figure S2). Thus, the nuclear and cytoplasmic positivity for each marker was given by the ratio between the number of positively stained neurons and the total number of neurons (estimated). Staining intensity was assessed in addition to positivity. For these two parameters (positivity and intensity), scores were defined as follows:
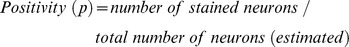

0 – no staining
1 – *p*≤Percentile 33% (P_0, 33_)
2 – P_0, 33_<*p*≤P_0, 66_
3 – *p*>P_0 66_

- Intensity of staining (*I*)

0 - no staining
1 - Weak.
2 - Weak - moderate
3 - Moderate
4 - Moderate - strong
5 - Strong

The expression value (E) of each marker for each sample was defined by the following equation:
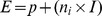
where

- *p* = positivity;
- n_i_ = number of intensity categories;
- *I* = intensity of staining.

Thus, we exclude the possibility of assigning the same expression values for samples with low positivity but high intensity as for samples with high positivity and low intensity.

### Statistical Analysis

Group differences in demographic variables were analyzed using analysis of variance (ANOVA) and χ-square tests as appropriate. Differences among medians of studied markers were assessed by Kruskal-Wallis tests, which were followed by Dunn’s test, using GraphPad Prism software version 6.0. A *P*-value of ≤0.05 was used as the criterion for statistical significance. Hierarchical clustering analysis was based on Pearson’ correlations and average linkage from expression values of assessed markers. Reliability of the clustering was assessed by the Bootstrap technique using MEV software [58].

## Results

In this study, markers were examined in the hippocampus, which is affected early in AD and which has been extensively studied. Our sample groups did not statistically differ in average age or in gender distribution, although the female frequency was higher in the CP-AD and P-AD groups. Significant differences in postmortem intervals (PMI) were also not detected among the groups (Table 3).

**Table 3:** Demographic statistics.

	CP-AD	P-AD	N	P-value
Age, mean (sd), y	86.1 (5.9)	85.3 (5.0)	84.6 (4.3)	0.595^a^
Women, n (%)	16 (84.2)	7 (61.5)	16 (51.6)	0.064^b^
PMI, mean (sd), h	15.0 (3.0)	13.6 (3.6)	14.4 (3.2)	0.516^a^

a ANOVA;

b χ-square; PMI, Postmortem Interval; CP-AD, clinical-pathological Alzheimer’s disease; P-AD, pathological Alzheimer’s disease; N, normal aging.

### Oxidative DNA Damage

**Figure 1:**
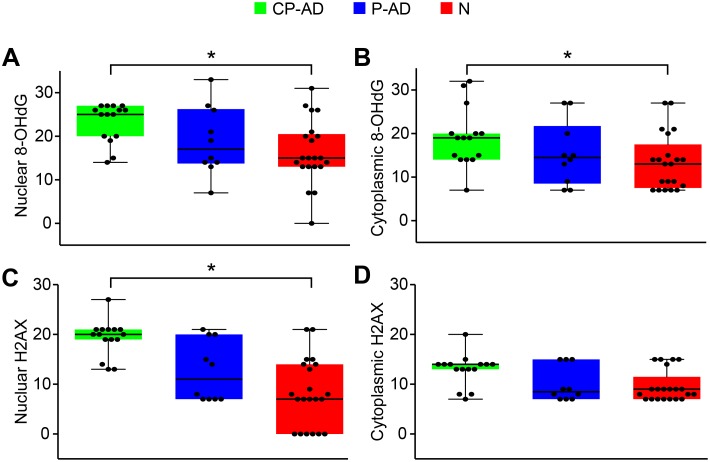
Expression levels of markers related to oxidative damage to DNA. Boxplots of 8-OHdG scores attributed to nuclear (A) and cytoplasmic (B) staining. Boxplots of H2AX scores attributed to nuclear (C) and cytoplasmic (D) staining. CP-AD, clinical-pathological Alzheimer’s disease (green boxes); P-AD, pathological Alzheimer’s disease (blue boxes); N, normal aging (red boxes).

Oxidative damage to DNA can be caused by reactive oxygen species (ROS), which are produced by radiation or by products of aerobic metabolism. The oxidized base, 8-Hydroxyguanine (8-OHdG), the most commonly analyzed biomarker of DNA damage, showed high levels of nuclear staining in all groups. However, a statistically significant difference was only found between CP-AD and N, with greater expression in demented subjects (Figure 1A; Table 4; Figure S3A, S3B, and S3C). A similar expression pattern was detected in the cytoplasmic compartment, where higher levels of 8-OHdG were found in CP-AD than N (Figure 1B; Table 5; Figure S3D, S3E, and S3F).

**Table 4:** Summary of nuclear expression of studied markers.

	Group medians (range)				Dunn’s multiple comparisons test *p* value		
	CP-AD	P-AD	N	Kruskal-Wallis *p* value	CP-AD vs. N	CP-AD vs. P-AD	P-AD vs. N
8-OHdG	25 (14–27)	17 (7–33)	15 (0–31)	0.0182*	0.0145*	0.3317	>0.9999
H2AX	20 (13–27)	11 (7–21)	07 (0–21)	0.0002*	0.0001*	0.0803	0.6308
p53	07 (0–15)	14 (7–21)	15 (7–21)	0.0001*	<0.0001*	0.0222*	>0.9999
PTEN	25 (19–27)	31 (25–33)	27 (19–33)	0.0002*	0.0093*	0.0003*	0.3738
BRCA1	19 (0–26)	25 (19–33)	21 (7–33)	0.0299*	0.1818	0.0341*	0.9349
ppAKT	0 (0–14)	0 (0–13)	0 (0–20)	0.4605	–	–	–
Cdk4	14.5 (0–27)	0 (0–19)	0 (0–21)	<0.0001*	<0.0001*	0.0047*	>0.9999
Cyclin D	15 (0–32)	7 (0–19)	0 (0–19)	<0.0001*	<0.0001*	0.0201*	0.2434
ppRB	26.5 (7–33)	14.5 (7–21)	13 (0–25)	<0.0001*	<0.0001*	0.0028*	>0.9999
E2F1	19 (0–26)	0 (0–19)	0 (0–15)	<0.0001*	<0.0001*	0.0058*	0.9468
Cdk1	20 (9–27)	3.5 (0–20)	0 (0–31)	<0.0001*	0.0002*	0.0005*	>0.9999
Cyclin B	15 (14–27)	10.5 (0–21)	7 (0–14)	<0.0001*	<0.0001*	0.0131*	0.2444
p27	0 (0–14)	14 (0–19)	9 (0–31)	0.0002*	0.0008*	0.0022*	>0.9999
Cdk5	7 (0–15)	14 (7–21)	15 (0–27)	0.001*	0.0015*	0.0126*	>0.9999
Apoptosis	20 (7–32)	3.5 (0–21)	0 (0–31)	<0.0001*	<0.0001*	0.0134*	0.9803

Expression values of nuclear staining are shown as median (min–max). Kruskal-Wallis tests were used for comparisons among the three groups (CP-AD, P-AD and N). Two-by-two group comparisons were performed by utilizing Dunn’s multiple comparisons tests. *indicates statistical significance (*P*≤0.05). CP-AD, clinical-pathological Alzheimer’s disease; P-AD, pathological Alzheimer’s disease; N, normal aging.

**Table 5:** Summary of cytoplasmic expression of studied markers.

	Group medians (range)				Dunn’s multiple comparisons test *p* value		
	CP-AD	P-AD	N	Kruskal-Wallis *p* value	CP-AD vs. N	CP-AD vs. P-AD	P-AD vs. N
8-OHdG	19 (7–32)	14.5 (7–27)	13 (7–27)	0.05*	0.0446*	0.5628	>0.9999
H2AX	14 (7–20)	8.5 (7–15)	9 (7–15)	0.1004	–	–	–
p53	7 (0–15)	7.5 (0–13)	0 (0–9)	0.4988	–	–	–
PTEN	0 (0–0)	0 (0–0)	0 (0–8)	0.2857	–	–	–
BRCA1	19 (0–32)	19 (0–33)	13.5 (0–27)	0.1381	–	–	–
ppAKT	8 (0–14)	14 (7–15)	13.5 (7–19)	0.025*	0.05*	0.05*	>0.9999
Cdk4	13 (0–15)	9 (7–21)	7 (0–20)	0.0051*	0.0083*	>0.9999	0.0898
Cyclin D	7 (0–14)	9 (0–15)	0 (0–15)	0.0072*	0.3813	0.269	0.0056*
ppRB	26.5 (0–33)	8.5 (0–26)	0 (0–31)	<0.0001*	<0.0001*	0.0271*	>0.9999
E2F1	0 (0–20)	7 (0–15)	7 (0–19)	0.8878	–	–	–
Cdk1	14 (7–21)	8.5 (7–19)	8 (0–19)	0.0468*	0.0467*	0.3132	>0.9999
Cyclin B	14 (7–21)	11 (7–15)	9 (0–19)	0.2904	–	–	–
p27	0 (0–0)	0 (0–9)	0 (0–13)	0.249	–	–	–
Cdk5	9 (0–15)	7 (0–14)	7 (0–19)	0.0949	–	–	–

Expression values of cytoplasmic staining are shown as median (min–max). Kruskal-Wallis tests were used for comparisons among the three groups (CP-AD, P-AD and N). Two-by-two group comparisons were performed by utilizing Dunn’s multiple comparisons tests. *indicates statistical significance (*P*≤0.05). CP-AD, clinical-pathological Alzheimer’s disease; P-AD, pathological Alzheimer’s disease; N, normal aging.

Another biomarker for DNA damage is H2AX, a histone that is phosphorylated in response to double-strand breaks of DNA, giving rise to λ-H2AX. In our samples, nuclear λ-H2AX expression was higher in CP-AD individuals than in normal aging group (N), whereas no significant difference in cytoplasmic expression was detected among the groups (Figure 1C and 1D; Tables 4 and 5; Figure S4).

### DNA Repair

High levels of oxidative damage can be caused by relatively inefficient DNA repair. Thus, we assayed some proteins involved in the DNA-damage repair system, whose roles are essential for detecting damage and triggering a repair response.

**Figure 2:**
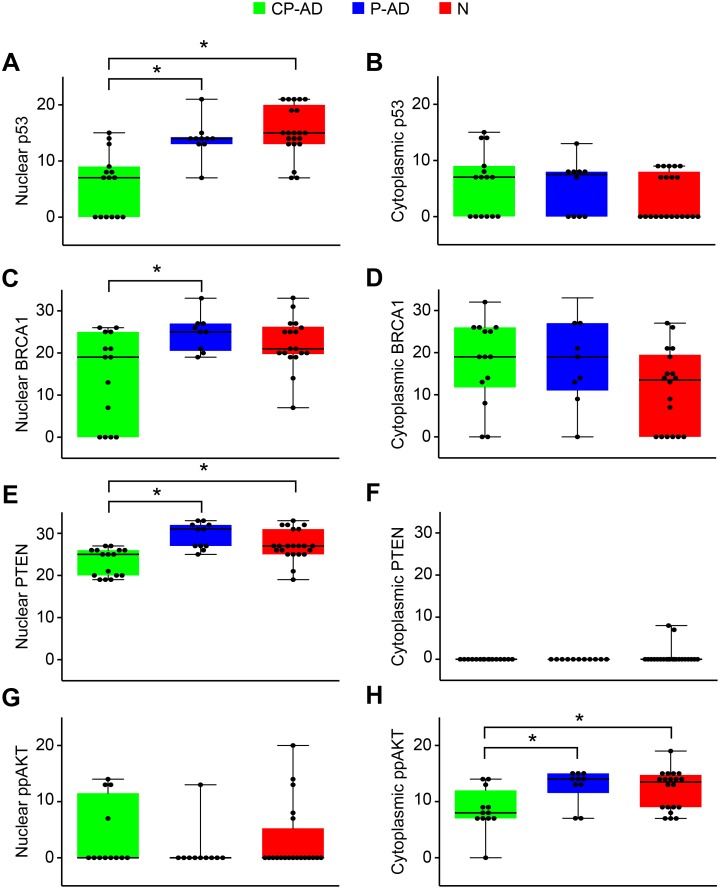
Expression levels of markers related to DNA repair. Boxplots of p53 scores attributed to nuclear (A) and cytoplasmic (B) staining. Boxplots of BRCA1 scores attributed to nuclear (C) and cytoplasmic (D) staining. Boxplots of PTEN scores attributed to nuclear staining (E) and cytoplasmic (F) staining. Boxplots of phospho-AKT scores attributed to nuclear (G) and cytoplasmic (H) staining. CP-AD, clinical-pathological Alzheimer’s disease (green boxes); P-AD, pathological Alzheimer’s disease (blue boxes); N, normal aging (red boxes).

p53 is a protein activated in response to a wide variety of stresses that can damage the cell genome; once it is bound to sites of DNA damage, p53 promotes DNA repair and simultaneously stimulates the transcription of direct effectors of cell-cycle arrest. Our results show that nuclear p53 expression was significantly greater in the P-AD and N groups than in the CP-AD group (Figure 2A; Table 4; Figure S5A, S5B, and S5C); however, for cytoplasmic expression, there were no significant differences among the groups (Figure 2B; Table 5; Figure S5D, S5E, and S5F).

BRCA1 is known to regulate transcription and cell-cycle progression; thus, the presence of BRCA1 is indicative of cell-cycle changes and DNA damage, both of which are pathogenic changes in AD. Significantly higher levels of nuclear BRCA1 were detected in P-AD individuals compared with CP-AD subjects, but differences were not significant either between P-AD and N or between CP-AD and N (Figure 2C; Table 4; Figure S6A, S6B, and S6C). For cytoplasmic staining, although higher expression was measured in CP-AD and P-AD individuals, no significant difference was found among the groups (Figure 2D; Table 5; Figure S6D, S6E, and S6F).

In the cytoplasm, PTEN antagonizes the phosphatidylinositol 3-kinase (PI3K/AKT) signaling pathway. PTEN also accumulates in the nucleus, where it has been shown to control DNA repair. In the nucleus, PTEN showed higher levels of expression in P-AD and N groups than in the CP-AD group (Figure 2E; Table 4; Figure S7A, S7B, and S7C). Unlike nuclear staining, cytoplasmic expression of PTEN was generally undetectable (Figure 2F; Table 5; Figure S7D, S7E, and S7F). PTEN acts as an endogenous inhibitor of AKT signaling. Although we found no cytoplasmic expression of PTEN, we found higher cytoplasmic expression of phospho-AKT in P-AD and N individuals than in CP-AD subjects (Figure 2G and 2H; Tables 4 and 5; Figure S8).

### Cell-cycle and Cell Death

To systematically evaluate the cell-cycle events in our groups, cell-cycle markers of early stages (G1 phase – Cdk4, cyclin D1, Cdk5; G1/S transition – phospho-Rb, E2F1) and later stages (G2/M phase – Cdk1, cyclin B1) were used in addition to the inhibitor of cyclin-dependent kinases (p27).

**Figure 3:**
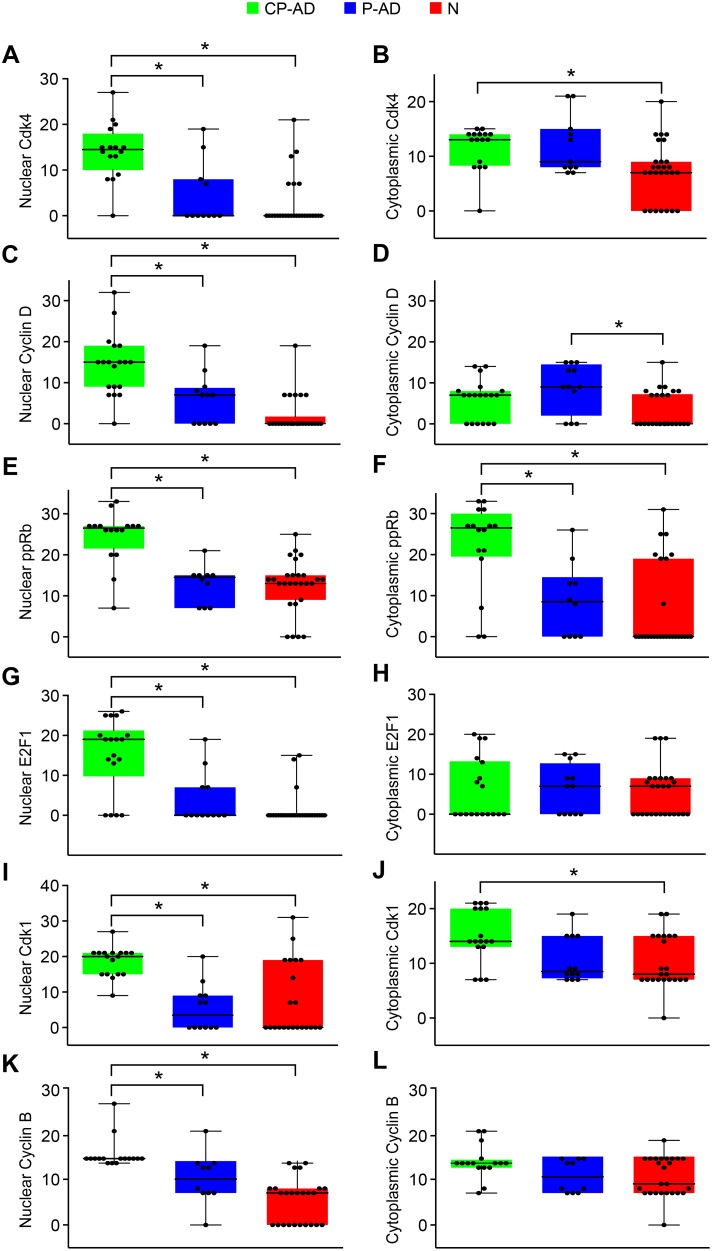
Expression levels of markers related to progression of cell cycle. Boxplots of Cdk4 scores attributed to nuclear (A) and cytoplasmic (B) staining. Boxplots of cyclin D scores attributed to nuclear (C) and cytoplasmic (D) staining. Boxplots of phospho-Rb scores attributed to nuclear (E) and cytoplasmic (F). Boxplots of E2F1 scores attributed to nuclear (G) and cytoplasmic (H) staining. Boxplots of Cdk1 scores attributed to nuclear (I) and cytoplasmic (J) staining. Boxplots of cyclin B scores attributed to nuclear (K) and cytoplasmic (L) staining. CP-AD, clinical-pathological Alzheimer’s disease (green boxes); P-AD, pathological Alzheimer’s disease (blue boxes); N, normal aging (red boxes).

**Figure 4:**
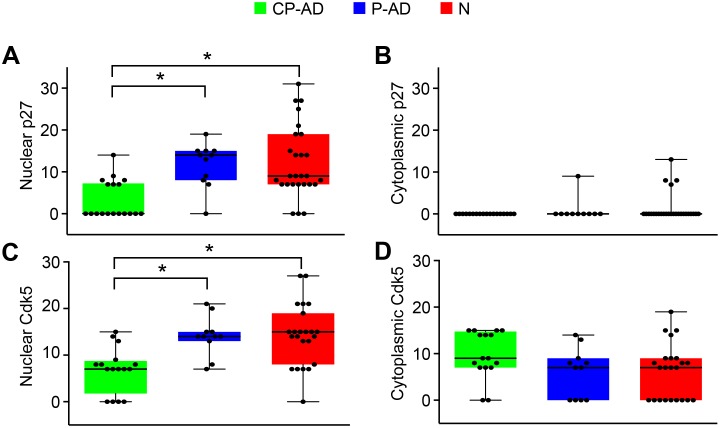
Expression levels of markers related to cell-cycle inhibition. Boxplots of p27 scores attributed to nuclear (A) and cytoplasmic (B) staining. Boxplots of Cdk5 scores attributed to nuclear (C) and cytoplasmic (D) staining. CP-AD, clinical-pathological Alzheimer’s disease (green boxes); P-AD, pathological Alzheimer’s disease (blue boxes); N, normal aging (red boxes).

Regarding nuclear staining, pyramidal neurons from the hippocampus of CP-AD individuals demonstrated higher expression levels of Cdk4, cyclin D, phospho-Rb, E2F1, Cdk1, and cyclin B than did those from either P-AD or N subjects (Figure 3A, 3C, 3E, 3G, 3I, and 3K; Table 4; Figures S9–S14). For the inhibitors of cell-cycle progression, Cdk5 and p27, the expression levels were lower in CP-AD individuals than in P-AD and N subjects (Figure 4A and 4C; Table 4; Figures S15 and S16).

For the cytoplasmic staining (Figure 3B, 3D, 3F, 3H, 3J, and 3L; Table 5; Figures S9–S14), significant differences were identified for Cdk4, phospho-Rb, and Cdk1 between CP-AD and N groups (with higher levels in CP-AD). Only cyclin D was different between the P-AD and N groups (higher level in P-AD). Cdk5 and p27 did not show significant differences among the three groups (Figure 4B and 4D; Table 5; Figures S15 and S16).

**Figure 5:**
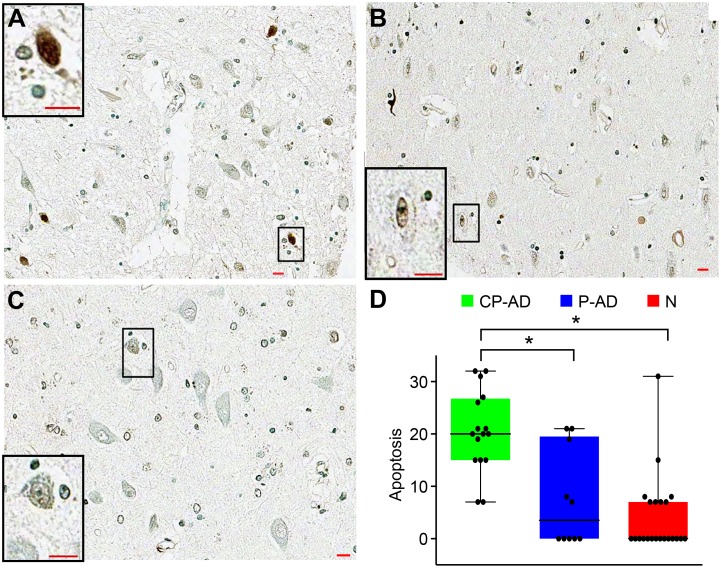
Staining patterns and expression levels of apoptosis marker. TUNEL staining of neurons is stronger in CP-AD (A) than in P-AD (B) or N (C). Boxplots of apoptosis scores (D). CP-AD, clinical-pathological Alzheimer’s disease (green boxes); P-AD, pathological Alzheimer’s disease (blue boxes); N, normal aging (red boxes).

To evaluate the neuronal cell death, terminal deoxynucleotidyltransferase-mediated biotinylated UTP nick end labeling (TUNEL) was performed *in situ*. Apoptotic neurons were found at higher rates in hippocampus from individuals with CP-AD than in samples from P-AD and N subjects, as can be seen in Figure 5 and Table 4.

An overview of the studied biomarkers is presented in Tables 4 and 5, wherein we can observe the following:

1. Markers of oxidative damage to DNA (8-OHdG and H2AX) were elevated in the CP-AD group compared to the N group, regarding both nuclear and cytoplasmic expression.
2. Markers of DNA repair (p53 and PTEN) were decreased in the CP-AD group compared to both the P-AD and N groups, regarding nuclear expression. BRCA1 was significantly decreased only in CP-AD compared to P-AD, although there is a trend of normal individuals showing similarity to preclinical subjects.
3. Markers of cell-cycle progression (Cdk4, cyclin D, phospho-Rb, E2F1, Cdk1, and cyclin B) were elevated in the CP-AD group compared to both the P-AD and N groups, regarding nuclear expression; some markers, such as Cdk4, phospho-Rb, and Cdk1, were found elevated in the CP-AD group compared to the N group for cytoplasmic expression.
4. Markers of cell-cycle inhibition (Cdk5, and p27) were decreased in the CP-AD group compared to both the P-AD and N groups, regarding nuclear expression.
5. Neuronal cell death was elevated in the CP-AD group compared to both the P-AD and N groups.

### Hierarchical Clustering

**Figure 6:**
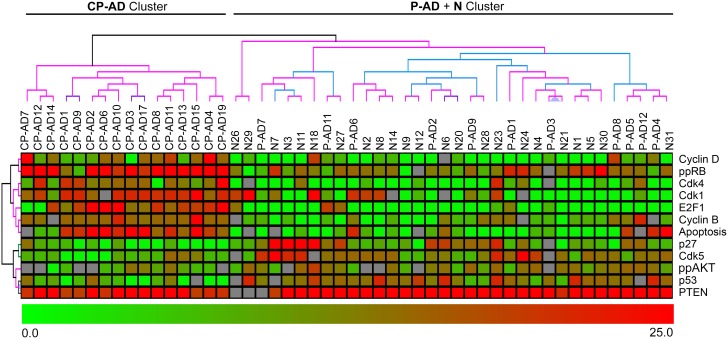
Hierarchical clustering analysis of CP-AD, P-AD and N samples. Hierarchical clustering was performed by using the expression values from the markers with differential expression between CP-AD and P-AD/N. Each row represents a single marker, and each column represents a sample. Red indicates high expression, and green indicates low expression. The cluster support was performed with the Bootstrap technique (black, 100% support; grey, 90–100%; blue, 80–90%; green, 70–80%; light yellow, 60–70%; dark yellow, 50–60%; magenta, 0–50%, red, 0%). CP-AD, clinical-pathological Alzheimer’s disease; P-AD, pathological Alzheimer’s disease; N, normal aging.

Searching for an expression pattern based on markers that achieved statistical significance between the CP-AD group and the other two groups (P-AD and N), a hierarchical clustering was conducted with all individuals in this study. The dendrogram in Figure 6 shows a sample discrimination pattern, with 100% support, in two clusters: 1) CP-AD cluster, which grouped all CP-AD samples; and 2) P-AD + N cluster, which grouped individuals with P-AD and individuals with normal aging.

This clustering displays an expression pattern composed of high levels of cell-cycle progression markers (Cyclin D, ppRb, Cdk4, Cdk1, E2F1, Cyclin B) and apoptosis markers, along with low levels of cell-cycle inhibitors and DNA repair markers (p27, Cdk5, ppAKT, p53, PTEN) in CP-AD individuals; P-AD and N individuals show the opposite pattern, with low levels of cell-cycle progression markers and apoptosis markers and high levels of cell-cycle inhibitors and DNA repair markers.

## Discussion

Individuals who have evidence of abundant AD pathology but do not meet clinical criteria for mild cognitive impairment (MCI) or dementia, thus preserving their cognitive functioning, have been described by a range of allied concepts such as “pathological aging”, “preclinical AD”, “asymptomatic AD”, and “resilient AD” [24], [59]–[62]. These individuals should possess resilience factors that protect them against significant accumulations of brain pathology. Therefore, understanding the molecular bases of these protection factors may help elucidate the expression of AD symptoms.

The etiology and pathogenesis of AD is not fully understood, but several factors have been implicated in the progression of AD [63]. Among these factors, oxidative stress has been shown to be involved in AD pathogenesis [64], [65]. We observed that biomarkers of oxidative damage in DNA are highly expressed in all groups in this study, which is expected because our population has an average age of 85 and accumulation of such damages occurs during aging [66]. We observed that 8-OHdG levels are significantly higher in CP-AD individuals than in subjects with normal aging (N), as demonstrated by others in hippocampus utilizing immunohistochemistry [67] and in various cortical regions utilizing other methodologies [26], [68]–[71]. Although we did not find significant differences in 8-OHdG between P-AD and CP-AD or between P-AD and N using immunohistochemistry, Lovell et al. [72] found a significant increase of hippocampal 8-OHdG immunofluorescence in “preclinical AD” individuals compared to normal controls. Regarding H2AX, its expression follows the same pattern as 8-OHdG, with significant elevation in CP-AD compared to N individuals. There are few studies examining H2AX in AD. One shows elevated H2AX in hippocampal astrocytes from AD patients (with dementia) in relation to normal controls [73]; another finds no difference in astrocytes or neurons in relation to AD progression [74]. Some authors have reasoned that the presence of DNA breaks by oxidative damage could be influenced by the post-mortem interval, but the presence of such an effect has not been clearly demonstrated [75]–[78]. PMI effects on DNA damage would most likely appear after long periods, depending on the brain area studied. Because no significant difference in PMI was detected among the groups, nor was there a significant difference in age, and our PMIs were not greater than 24 hours, we consider it unlikely that the PMI would significantly interfere in our DNA damage findings.

Elevated levels of oxidative stress markers and mitochondrial dysfunction have been found in AD. Recent studies have reported alterations in DNA repair proteins (cell-cycle checkpoint proteins and tumor suppressors) of pathways involved in the repair of oxidative stress-induced lesions in patients with AD [28], [79]. It is generally accepted that accumulation of DNA damage and the impairment of its repair mechanism is a prominent feature of aging in the CNS [80], and the impairment of this process is believed to be exacerbated in dementia and AD [81]–[83]. Among the studied biomarkers, BRCA1 is associated with a spectrum of functions related to the preservation of genome stability, such as the repair of oxidative damage to DNA [84], [85]. Therefore, its presence suggests alterations in the cell-cycle and DNA damage, both changes that are featured in AD. In our analysis of nuclear BRCA1 expression, we found a significant elevation in P-AD compared with CP-AD individuals. Thus, we speculate that in the face of AD pathology, individuals with increased activation of this repair system may have a compensatory ability to maintain brain homeostasis and reduce impairments in cognitive function. In studying cytoplasmic expression, Evans et al. [33] found elevated levels of BRCA1 in individuals with AD in addition to showing that BRCA1 was co-located with neurofibrillary tangles. Interestingly, our results showed higher levels of cytoplasmic BRCA1 in the cases with AD pathology (CP-AD and P-AD) compared to the normal group, although no statistically significant difference has been shown.

p53 is a tumor-suppressor protein that is activated in response to a variety of stressors that can damage the integrity of the cellular genome [86]. PTEN may have functions in the maintenance of genomic stability mediated at least in part by PI3K-independent mechanisms [87], [88]. Under oxidative stress, PTEN accumulates in the nucleus and can bind to p53, amplifying its function [89], [90]. Furthermore, Bassiet et al. [91] demonstrated that nuclear PTEN is involved in DNA repair, and cells without nuclear PTEN are more sensitive to DNA damage. Griffin et al. [32] showed a decrease in nuclear PTEN immunoreactivity in AD neurons from the hippocampus and entorhinal cortex. Sonoda et al. [31] observed a decrease in nuclear and cytoplasmic staining in brains of Alzheimer’s patients compared to brains of individuals with normal aging. We also demonstrate a lower expression level of PTEN in the nuclei of hippocampal neurons from individuals with CP-AD compared to both P-AD and N subjects. Thus, the nuclear downregulation of both p53 and PTEN found in demented individuals in relation to nondemented subjects suggests that DNA repair may play a role in the symptom expression in AD.

In the cytoplasm, PTEN acts as a specific phosphatase for 3′-phosphatidylinositol and is the major negative regulator of the activity of the phosphatidylinositol-3-kinase (PI3K) signaling pathway [92]. Levels of Akt phosphorylation can influence the location of PTEN. If Akt phosphorylation increases, PTEN is exported to the cytoplasm, inhibiting PIP3 formation and thereby inhibiting downstream pathways. If Akt is not phosphorylated, GSK3-β remains active [93] and is described as responsible for events related to AD pathology, including the hyperphosphorylation of Tau [94]. Although we did not observe cytoplasmic PTEN expression, we demonstrated reduced levels of phosphorylated Akt in the cytoplasm in CP-AD group compared to the P-AD and N groups. Notably, phosphorylated Akt may enhance cell survival by blocking the function of proteins and apoptotic processes [95].

There is also growing evidence for the association between DNA damage and increased expression of cell-cycle markers in AD [34], [38]. Although it is well established that neurons in the adult brain are post-mitotic - except in the hippocampal dentate gyrus and the subventricular zone [96] - neuropathological features of AD, such as the accumulation of Aβ and tau, have been shown to be associated with re-entry into the cell-cycle [47], [97]. In fact, the re-expression of cell-cycle proteins in neurons can be induced by treatment with Aβ and/or tau protein [97], [98]. Furthermore, studies have demonstrated an association between Aβ treatment/accumulation and neuronal cell death [99]–[101]. Previous immunohistochemical studies are consistent with our findings concerning the comparison between AD individuals (with dementia, here called CP-AD) and control individuals (here called N). For instance: 1) our findings that cell-cycle-progression regulators, such as Cdk4, Cdk1, cyclin D, cyclin B, phospho-Rb, and E2F1, are upregulated in CP-AD compared with N are consistent with other studies that indicate that cell-cycle molecules are upregulated in AD neurons [34], [37], [40], [41], [46], [102]; 2) our finding that the cell-cycle inhibitor p27 is downregulated in CP-AD when compared with N, is corroborated by Ogawa et al. [39], who demonstrated a lower level of p27 expression in the nucleus of AD neurons. Cdk5 is another molecule for which there was little evidence indicating a role in cell-cycle regulation, but recent data have suggested a crucial role for Cdk5 as a cell-cycle suppressor in post-mitotic neurons. Studies have proposed that Cdk5 nuclear localization relies on its interaction with p27, and its cell-cycle suppression activity is achieved by direct binding to E2F1, blocking the access of E2F1 to the DNA and thus avoiding the transcription of genes responsible for cell-cycle progression [103], [104]. Our results are interesting because, in CP-AD subjects, we found decreased levels of nuclear Cdk5 and p27 and increased nuclear E2F1, suggesting a loss of the cell-cycle suppression function in demented individuals (CP-AD) compared to nondemented individuals (P-AD and N). Interestingly, in our P-AD group, all cell-cycle markers cited above showed expression levels similar to those in individuals with normal aging (N), indicating that, even in the presence of AD pathology, post-mitotic neurons from individuals with healthy cognition retain control of the cell-cycle.

To emphasize a potential role of cell-cycle regulation and DNA repair in the symptomatic progression of AD, Yang et al. [34] showed that in brains of people who died with MCI, the percentage of hippocampal neurons positive for cell-cycle proteins (PCNA, cyclin D and cyclin B) was very close to that found in individuals who died in advanced stages of AD (demented). Moreover, decreased activity of proteins involved with DNA repair has been reported in MCI subjects [105], [106]. Therefore, these findings, along with ours, suggest that the manifestation of cognitive decline could be associated with defects in cell-cycle regulation and DNA repair.

Cell-cycle studies have shown that ectopic expression of cell-cycle markers is associated with neuronal cell death [46]–[48]. The type of cell death suffered by neurons in AD is still a controversial issue, but it is clear that there is a progressive atrophy due to brain cell and synaptic losses. Neurodegeneration in AD can take several years, and neuronal death is not the result of a single acute insult, but is likely the consequence of many triggers that induce compensatory responses for a long period of time until there is a loss of the capacity to maintain homeostasis [107]. Some studies have reported preserved neuronal numbers and synaptic densities in the hippocampus in “preclinical/resilient AD” compared with normal and clinical AD cases [62], [108], [109]. Our findings are consistent with the literature, wherein a cell death rate in the P-AD group similar to the normal group indicates preserved neuronal cell numbers, whereas increased cell death expression in the CP-AD group suggests neuronal loss.

In general, we observed reduced levels of DNA repair and cell-cycle inhibition markers and elevated levels of cell-cycle progression markers in association with increased levels of cell death in post-mitotic neurons of clinical and pathological AD subjects. In contrast, individuals who have AD neuropathology but no evidence of cognitive impairment (P-AD) present an expression profile similar to that of individuals with normal aging - with opposite levels of those markers, suggesting that healthy cognition may be associated with preserved DNA damage repair and cell-cycle regulation. Therefore, this indicates that these individuals (P-AD) had reserve, repair or resilience factors that protected them against the deleterious effects of brain pathology, and we speculate that DNA repair, cell-cycle regulation and neuronal cell death are molecular factors that may contribute to the clinical expression of AD.

Although additional studies are required to better understand the pathogenic mechanisms, especially the early stages of AD, and to uncover and validate biomarkers of resilience, we believe that our findings are an important step in better understanding the molecular neurobiology of healthy brain aging even in the face of brain pathology (resilient brain aging) and may serve to generate possibilities for new treatment strategies.

## Supporting Information

Figure S1 **Schematic model to estimate the numbers of hippocampal neurons.** Pyramidal neurons from five randomly selected hippocampal cores (yellow balls) of three different slides were counted, and then the average number of counted neurons was used to estimate the total number of hippocampal neurons for further analysis.
(TIF)

Figure S2 **Counting of positively stained neurons.** Using the tool ‘Events’ of ZEN software, neurons were manually selected, considering the nuclear (blue markers) and cytoplasmic (green markers) staining separately. The software then gives the number of selected events (blue and green arrows).
(TIF)

Figure S3 **Hippocampus stained for 8-OHdG.** Immunohistochemical immunoreactivity of 8-OHdG in the nuclei (top) of hippocampal neurons from CP-AD (A), P-AD (B), and N (C) individuals. Immunohistochemical immunoreactivity of 8-OHdG in the cytoplasm (bottom) of hippocampal neurons from CP-AD (D), P-AD (E), and N (F) individuals. Larger dashed boxes show magnifications of the smaller boxes. CP-AD, clinical-pathological Alzheimer’s disease; P-AD, pathological Alzheimer’s disease; N, normal aging. Scale bars = 50 pixels.
(TIF)

Figure S4 **Hippocampus stained for λ-H2AX.** Immunohistochemical immunoreactivity of λ-H2AX in the nuclei (top) of hippocampal neurons from CP-AD (A), P-AD (B), and N (C) individuals. Immunohistochemical immunoreactivity of λ-H2AX in the cytoplasm (bottom) of hippocampal neurons from CP-AD (D), P-AD (E), and N (F) individuals. Larger dashed boxes show magnifications of the smaller boxes. CP-AD, clinical-pathological Alzheimer’s disease; P-AD, pathological Alzheimer’s disease; N, normal aging. Scale bars = 50 pixels.
(TIF)

Figure S5 **Hippocampus stained for p53.** Immunohistochemical immunoreactivity of p53 in the nucleus (top) of hippocampal neurons from CP-AD (A), P-AD (B), and N (C) individuals. Immunohistochemical immunoreactivity of p53 in the cytoplasm (bottom) of hippocampal neurons from CP-AD (D), P-AD (E), and N (F) individuals. Larger dashed boxes show magnifications of the smaller boxes. CP-AD, clinical-pathological Alzheimer’s disease; P-AD, pathological Alzheimer’s disease; N, normal aging. Scale bars = 50 pixels.
(TIF)

Figure S6 **Hippocampus stained for BRCA1.** Immunohistochemical immunoreactivity of BRCA1 in the nuclei (top) of hippocampal neurons from CP-AD (A), P-AD (B), and N (C) individuals. Immunohistochemical immunoreactivity of BRCA1 in the cytoplasm (bottom) of hippocampal neurons from CP-AD (D), P-AD (E), and N (F) individuals. Larger dashed boxes show magnifications of the smaller boxes. CP-AD, clinical-pathological Alzheimer’s disease; P-AD, pathological Alzheimer’s disease; N, normal aging. Scale bars = 50 pixels.
(TIF)

Figure S7 **Hippocampus stained for PTEN.** Immunohistochemical immunoreactivity of PTEN in the nuclei (top) of hippocampal neurons from CP-AD (A), P-AD (B), and N (C) individuals. Immunohistochemical immunoreactivity of PTEN in the cytoplasm (bottom) of hippocampal neurons from CP-AD (D), P-AD (E), and N (F) individuals. Larger dashed boxes show magnifications of the smaller boxes. CP-AD, clinical-pathological Alzheimer’s disease; P-AD, pathological Alzheimer’s disease; N, normal aging. Scale bars = 50 pixels.
(TIF)

Figure S8 **Hippocampus stained for phospho-AKT.** Immunohistochemical immunoreactivity of phospho-AKT in the nuclei (top) of hippocampal neurons from CP-AD (A), P-AD (B), and N (C) individuals. Immunohistochemical immunoreactivity of phospho-AKT in the cytoplasm (bottom) of hippocampal neurons from CP-AD (D), P-AD (E), and N (F) individuals. Larger dashed boxes show magnifications of the smaller boxes. CP-AD, clinical-pathological Alzheimer’s disease; P-AD, pathological Alzheimer’s disease; N, normal aging. Scale bars = 50 pixels.
(TIF)

Figure S9 **Hippocampus stained for Cdk4.** Immunohistochemical immunoreactivity of Cdk4 in the nuclei (top) of hippocampal neurons from CP-AD (A), P-AD (B), and N (C) individuals. Immunohistochemical immunoreactivity of Cdk4 in the cytoplasm (bottom) of hippocampal neurons from CP-AD (D), P-AD (E), and N (F) individuals. Larger dashed boxes show magnifications of the smaller boxes. CP-AD, clinical-pathological Alzheimer’s disease; P-AD, pathological Alzheimer’s disease; N, normal aging. Scale bars = 50 pixels.
(TIF)

Figure S10 **Hippocampus stained for Cyclin D.** Immunohistochemical immunoreactivity of cyclin D in the nuclei (top) of hippocampal neurons from CP-AD (A), P-AD (B), and N (C) individuals. Immunohistochemical immunoreactivity of cyclin D in the cytoplasm (bottom) of hippocampal neurons from CP-AD (D), P-AD (E), and N (F) individuals. Larger dashed boxes show magnifications of the smaller boxes. CP-AD, clinical-pathological Alzheimer’s disease; P-AD, pathological Alzheimer’s disease; N, normal aging. Scale bars = 50 pixels.
(TIF)

Figure S11 **Hippocampus stained for phospho-Rb.** Immunoreactivity of phospho-Rb in the nuclei (top) of hippocampal neurons from CP-AD (A), P-AD (B), and N (C) individuals. Immunoreactivity of phospho-Rb in the cytoplasm (bottom) of hippocampal neurons from CP-AD (D), P-AD (E), and N (F) individuals. Larger dashed boxes show magnifications of the smaller boxes. CP-AD, clinical-pathological Alzheimer’s disease; P-AD, pathological Alzheimer’s disease; N, normal aging. Scale bars = 50 pixels.
(TIF)

Figure S12 **Hippocampus stained for E2F1.** Immunoreactivity of E2F1 in the nuclei (top) of hippocampal neurons from CP-AD (A), P-AD (B), and N (C) individuals. Immunoreactivity of E2F1 in the cytoplasm (bottom) of hippocampal neurons from CP-AD (D), P-AD (E), and N (F) individuals. Larger dashed boxes show magnifications of the smaller boxes. CP-AD, clinical-pathological Alzheimer’s disease; P-AD, pathological Alzheimer’s disease; N, normal aging. Scale bars = 50 pixels.
(TIF)

Figure S13 **Hippocampus stained for Cdk1.** Immunoreactivity of Cdk1 in the nuclei (top) of hippocampal neurons from CP-AD (A), P-AD (B), and N (C) individuals. Immunoreactivity of Cdk1 in the cytoplasm (bottom) of hippocampal neurons from CP-AD (D), P-AD (E), and N (F) individuals. Larger dashed boxes show magnifications of the smaller boxes. CP-AD, clinical-pathological Alzheimer’s disease; P-AD, pathological Alzheimer’s disease; N, normal aging. Scale bars = 50 pixels.
(TIF)

Figure S14 **Hippocampus stained for cyclin B.** Immunoreactivity of cyclin B in the nuclei (top) of hippocampal neurons from CP-AD (A), P-AD (B), and N (C) individuals. Immunoreactivity of cyclin B in the cytoplasm (bottom) of hippocampal neurons from CP-AD (D), P-AD (E), and N (F) individuals. Larger dashed boxes show magnifications of the smaller boxes. CP-AD, clinical-pathological Alzheimer’s disease; P-AD, pathological Alzheimer’s disease; N, normal aging. Scale bars = 50 pixels.
(TIF)

Figure S15 **Hippocampus stained for p27.** Immunoreactivity of p27 in the nuclei (top) of hippocampal neurons from CP-AD (A), P-AD (B), and N (C) individuals. Immunoreactivity of p27 in the cytoplasm (bottom) of hippocampal neurons from CP-AD (D), P-AD (E), and N (F) individuals. Larger dashed boxes show magnifications of the smaller boxes. CP-AD, clinical-pathological Alzheimer’s disease; P-AD, pathological Alzheimer’s disease; N, normal aging. Scale bars = 50 pixels.
(TIF)

Figure S16 **Hippocampus stained for Cdk5.** Immunoreactivity of Cdk5 in the nuclei (top) of hippocampal neurons from CP-AD (A), P-AD (B), and N (C) individuals. Immunoreactivity of Cdk5 in the cytoplasm (bottom) of hippocampal neurons from CP-AD (D), P-AD (E), and N (F) individuals. Larger dashed boxes show magnifications of the smaller boxes. CP-AD, clinical-pathological Alzheimer’s disease; P-AD, pathological Alzheimer’s disease; N, normal aging. Scale bars = 50 pixels.
(TIF)
