# Supplementary figures and images for: Repair of Oxidative DNA Damage, Cell-Cycle Regulation and Neuronal Death May Influence the Clinical Manifestation of Alzheimer’s Disease
Source: PLoS One. 2014 Jun 17;9(6):e99897. doi: 10.1371/journal.pone.0099897 (PMC4061071; doi:10.1371/journal.pone.0099897)

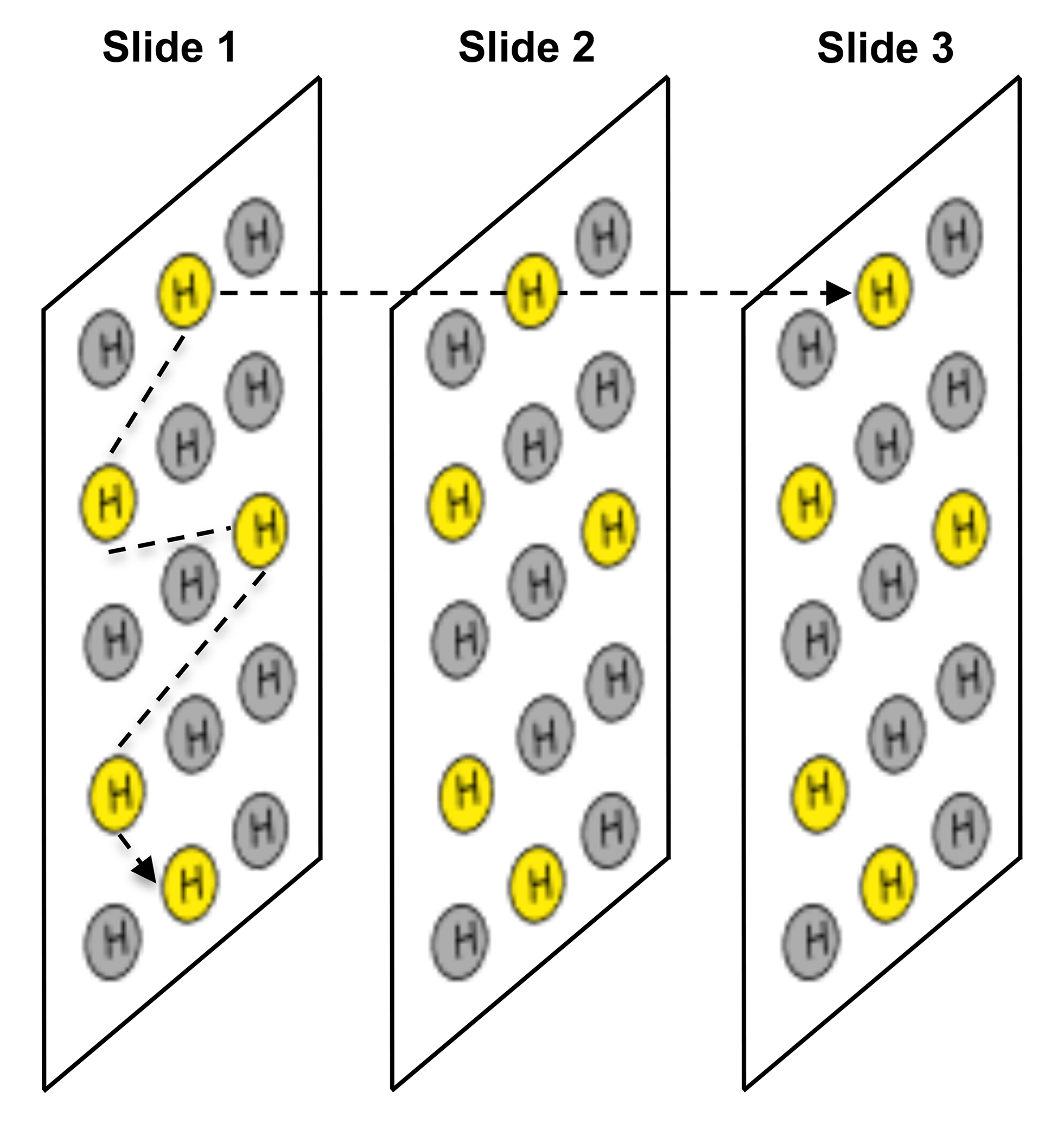

Supplement: Figure S1 — Schematic model to estimate the numbers of hippocampal neurons. Pyramidal neurons from five randomly selected hippocampal cores (yellow balls) of three different slides were counted, and then the average number of counted neurons was used to estimate the total number of hippocampal neurons for further analysis. (TIF) [file pone.0099897.s001.tif]

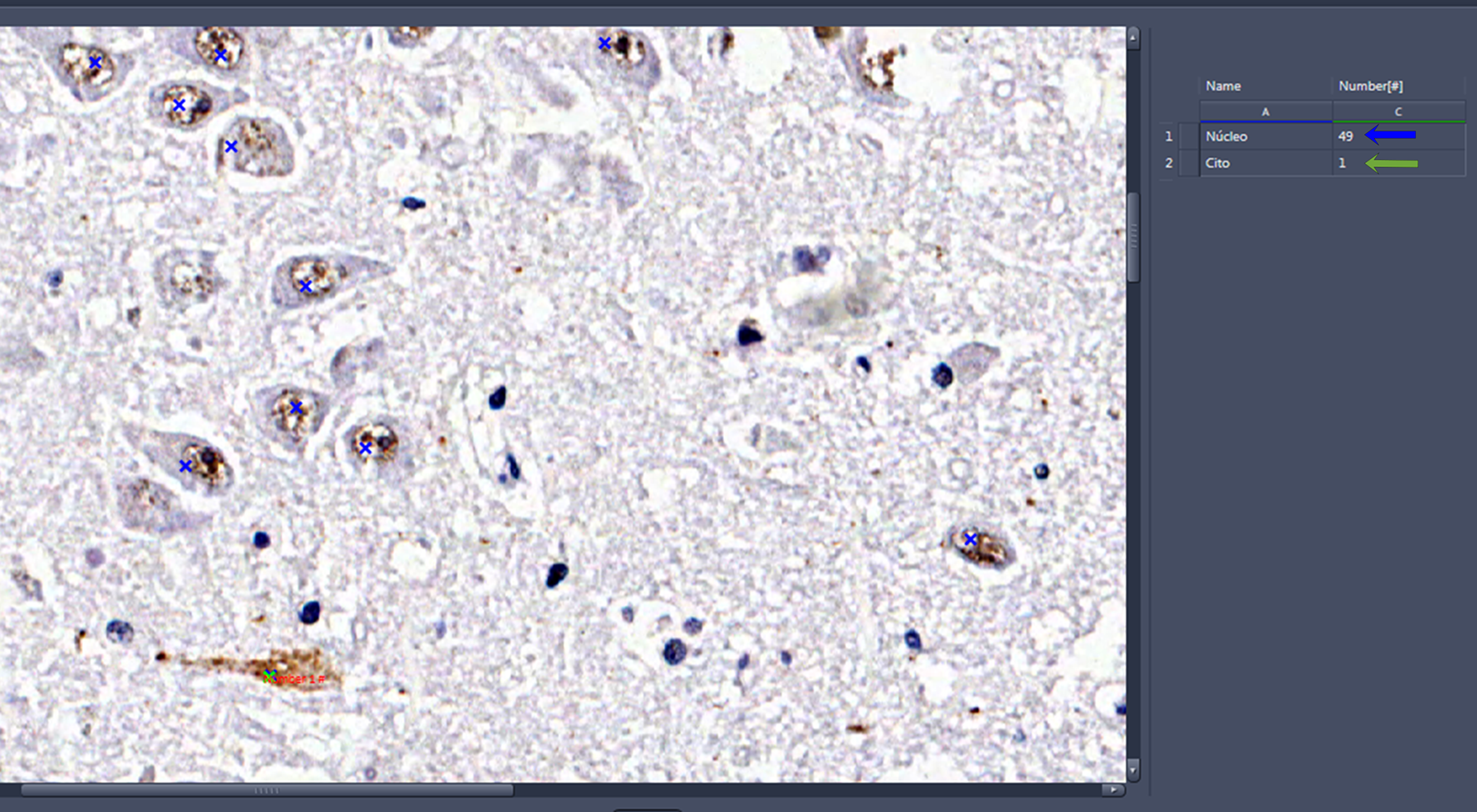

Supplement: Figure S2 — Counting of positively stained neurons. Using the tool ‘Events’ of ZEN software, neurons were manually selected, considering the nuclear (blue markers) and cytoplasmic (green markers) staining separately. The software then gives the number of selected events (blue and green arrows). (TIF) [file pone.0099897.s002.tif]

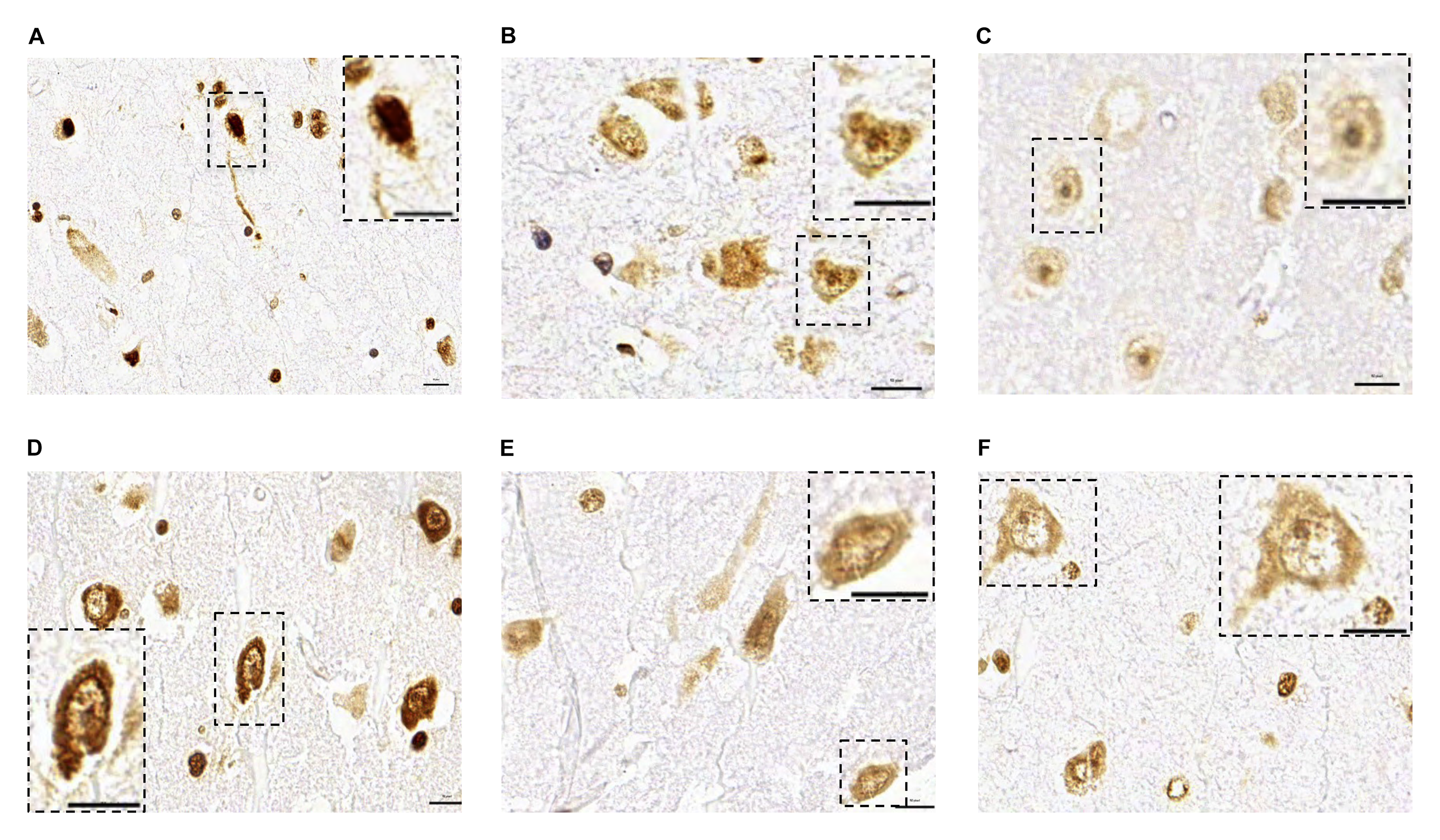

Supplement: Figure S3 — Hippocampus stained for 8-OHdG. Immunohistochemical immunoreactivity of 8-OHdG in the nuclei (top) of hippocampal neurons from CP-AD (A), P-AD (B), and N (C) individuals. Immunohistochemical immunoreactivity of 8-OHdG in the cytoplasm (bottom) of hippocampal neurons from CP-AD (D), P-AD (E), and N (F) individuals. Larger dashed boxes show magnifications of the smaller boxes. CP-AD, clinical-pathological Alzheimer’s disease; P-AD, pathological Alzheimer’s disease; N, normal aging. Scale bars = 50 pixels. (TIF) [file pone.0099897.s003.tif]

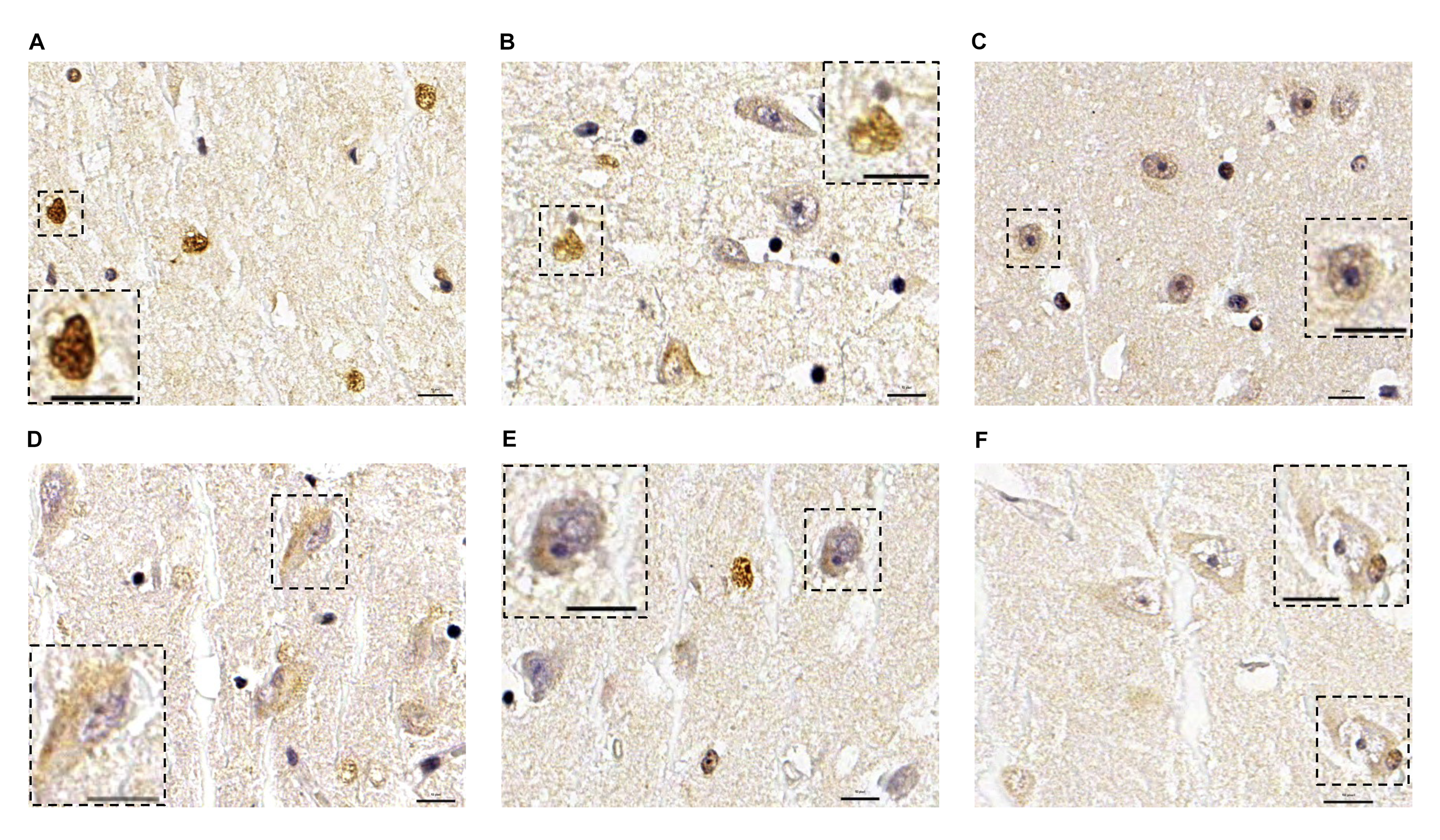

Supplement: Figure S4 — Hippocampus stained for λ-H2AX. Immunohistochemical immunoreactivity of λ-H2AX in the nuclei (top) of hippocampal neurons from CP-AD (A), P-AD (B), and N (C) individuals. Immunohistochemical immunoreactivity of λ-H2AX in the cytoplasm (bottom) of hippocampal neurons from CP-AD (D), P-AD (E), and N (F) individuals. Larger dashed boxes show magnifications of the smaller boxes. CP-AD, clinical-pathological Alzheimer’s disease; P-AD, pathological Alzheimer’s disease; N, normal aging. Scale bars = 50 pixels. (TIF) [file pone.0099897.s004.tif]

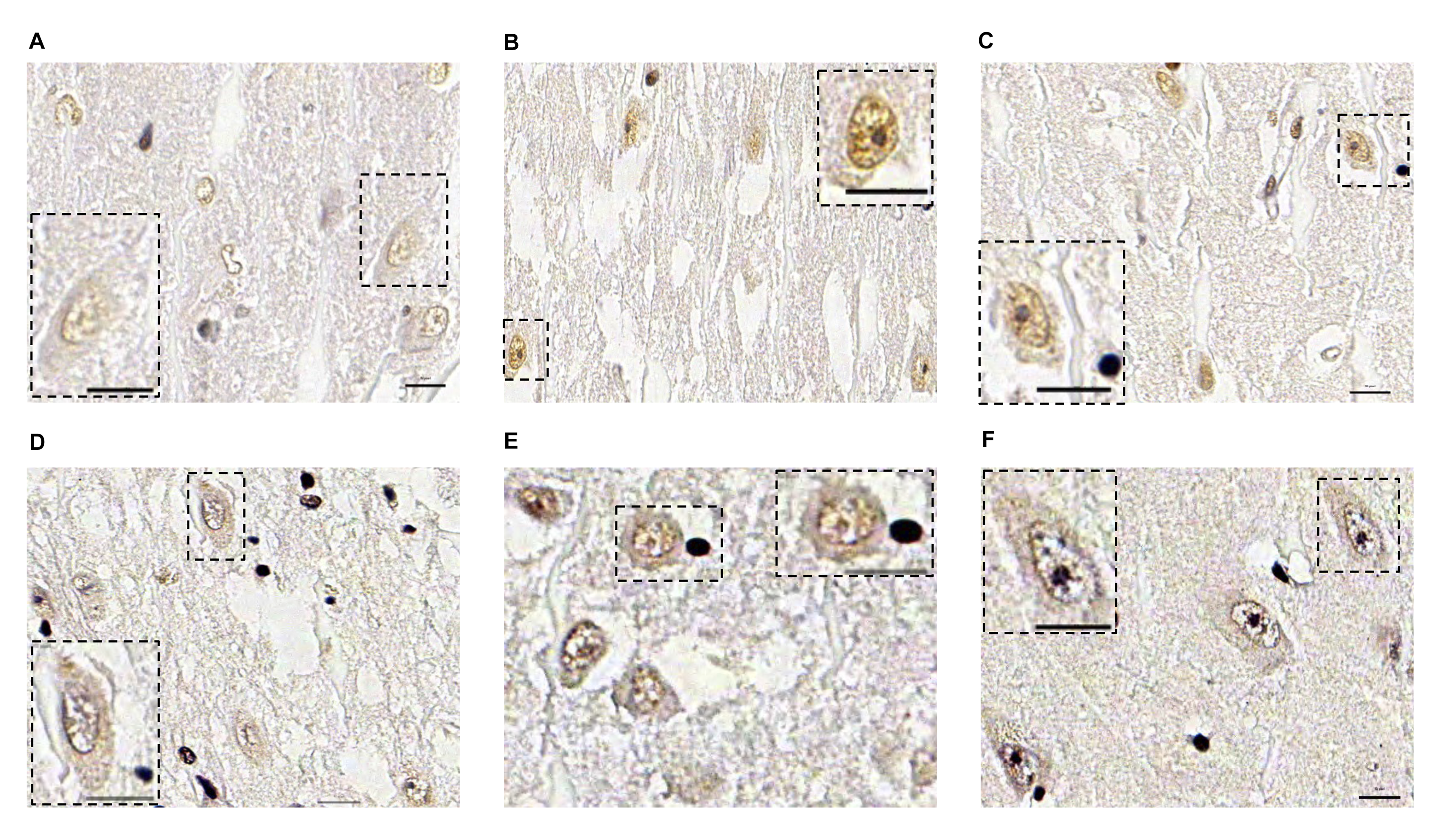

Supplement: Figure S5 — Hippocampus stained for p53. Immunohistochemical immunoreactivity of p53 in the nucleus (top) of hippocampal neurons from CP-AD (A), P-AD (B), and N (C) individuals. Immunohistochemical immunoreactivity of p53 in the cytoplasm (bottom) of hippocampal neurons from CP-AD (D), P-AD (E), and N (F) individuals. Larger dashed boxes show magnifications of the smaller boxes. CP-AD, clinical-pathological Alzheimer’s disease; P-AD, pathological Alzheimer’s disease; N, normal aging. Scale bars = 50 pixels. (TIF) [file pone.0099897.s005.tif]

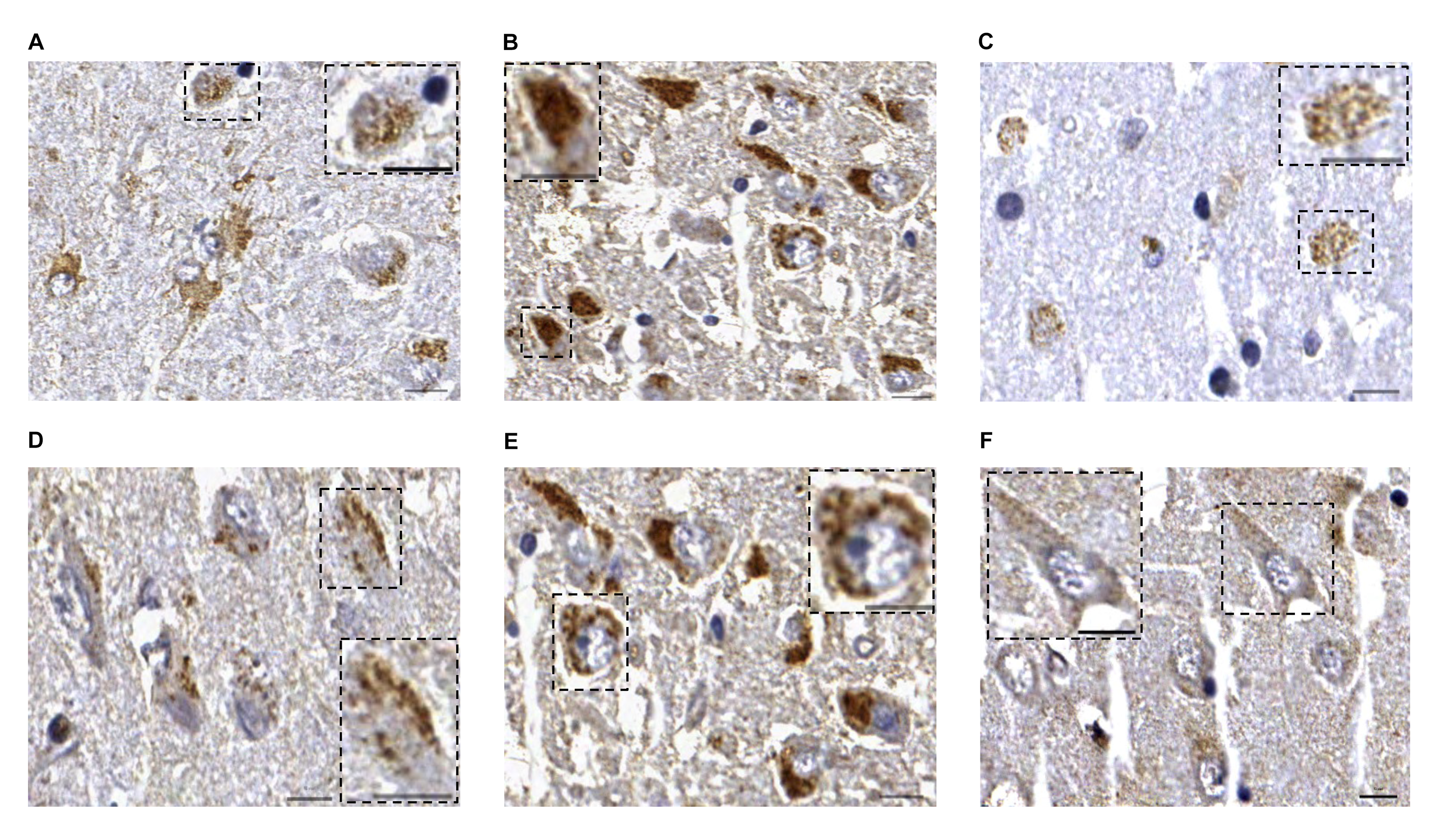

Supplement: Figure S6 — Hippocampus stained for BRCA1. Immunohistochemical immunoreactivity of BRCA1 in the nuclei (top) of hippocampal neurons from CP-AD (A), P-AD (B), and N (C) individuals. Immunohistochemical immunoreactivity of BRCA1 in the cytoplasm (bottom) of hippocampal neurons from CP-AD (D), P-AD (E), and N (F) individuals. Larger dashed boxes show magnifications of the smaller boxes. CP-AD, clinical-pathological Alzheimer’s disease; P-AD, pathological Alzheimer’s disease; N, normal aging. Scale bars = 50 pixels. (TIF) [file pone.0099897.s006.tif]

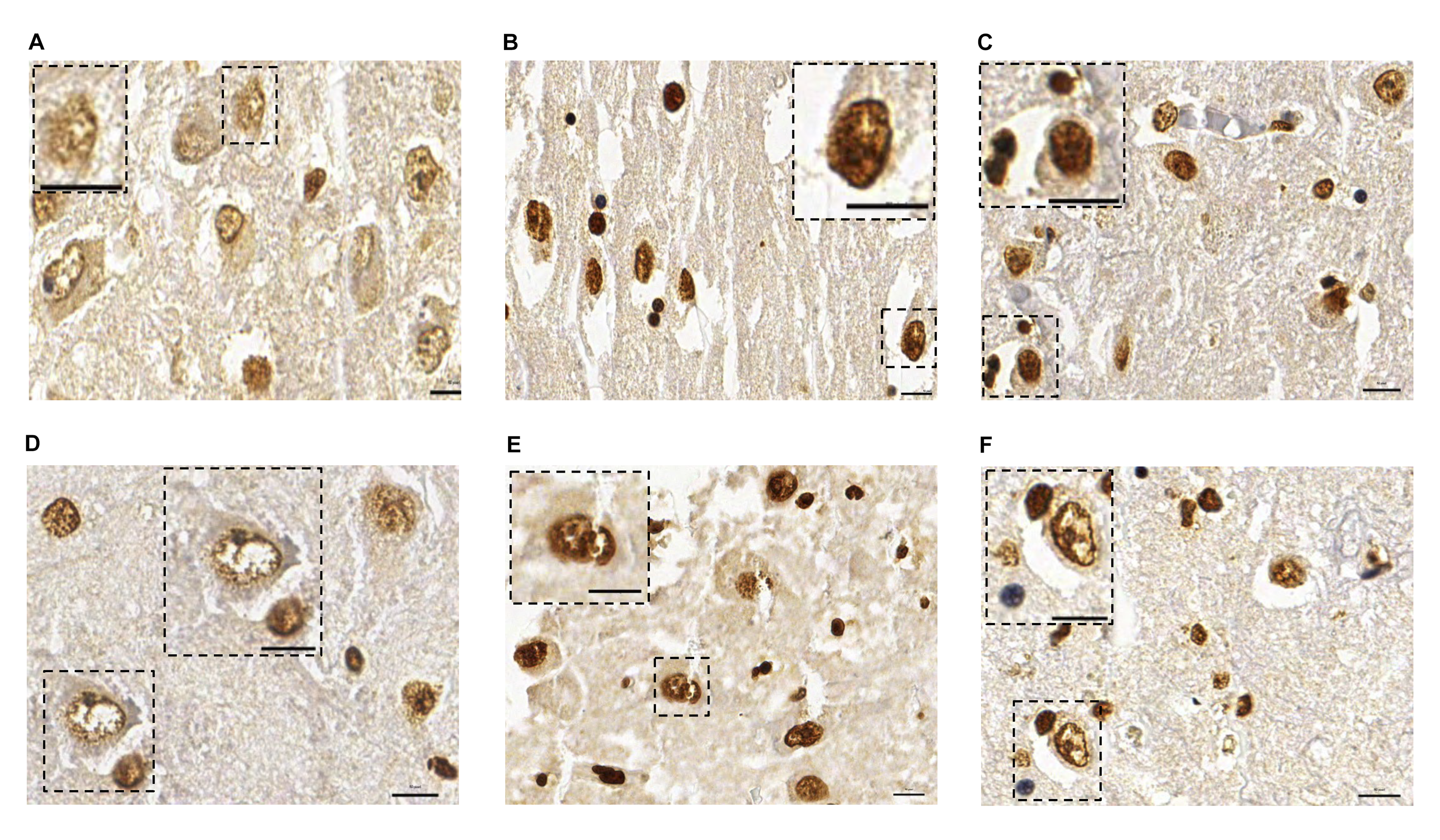

Supplement: Figure S7 — Hippocampus stained for PTEN. Immunohistochemical immunoreactivity of PTEN in the nuclei (top) of hippocampal neurons from CP-AD (A), P-AD (B), and N (C) individuals. Immunohistochemical immunoreactivity of PTEN in the cytoplasm (bottom) of hippocampal neurons from CP-AD (D), P-AD (E), and N (F) individuals. Larger dashed boxes show magnifications of the smaller boxes. CP-AD, clinical-pathological Alzheimer’s disease; P-AD, pathological Alzheimer’s disease; N, normal aging. Scale bars = 50 pixels. (TIF) [file pone.0099897.s007.tif]

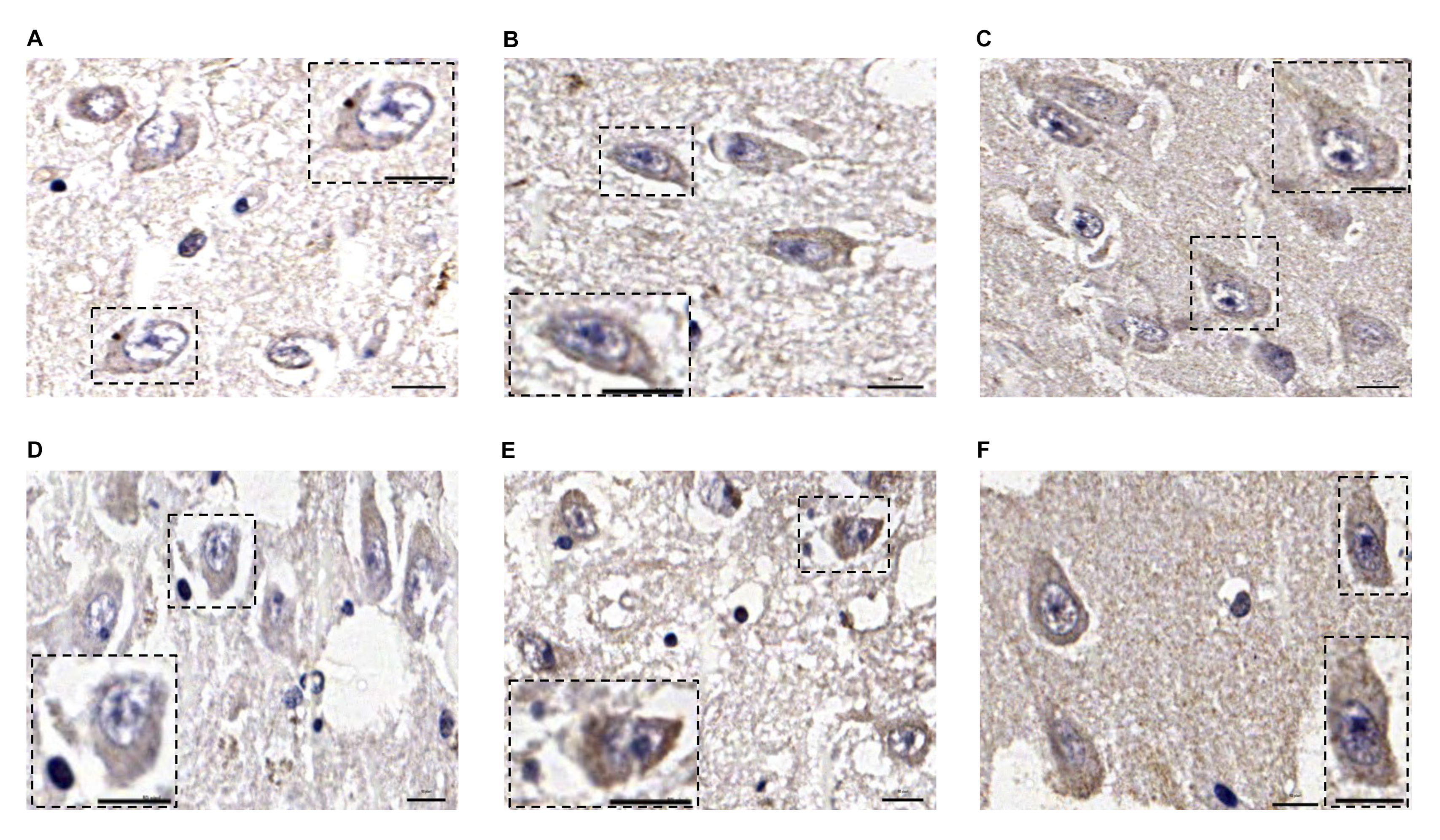

Supplement: Figure S8 — Hippocampus stained for phospho-AKT. Immunohistochemical immunoreactivity of phospho-AKT in the nuclei (top) of hippocampal neurons from CP-AD (A), P-AD (B), and N (C) individuals. Immunohistochemical immunoreactivity of phospho-AKT in the cytoplasm (bottom) of hippocampal neurons from CP-AD (D), P-AD (E), and N (F) individuals. Larger dashed boxes show magnifications of the smaller boxes. CP-AD, clinical-pathological Alzheimer’s disease; P-AD, pathological Alzheimer’s disease; N, normal aging. Scale bars = 50 pixels. (TIF) [file pone.0099897.s008.tif]

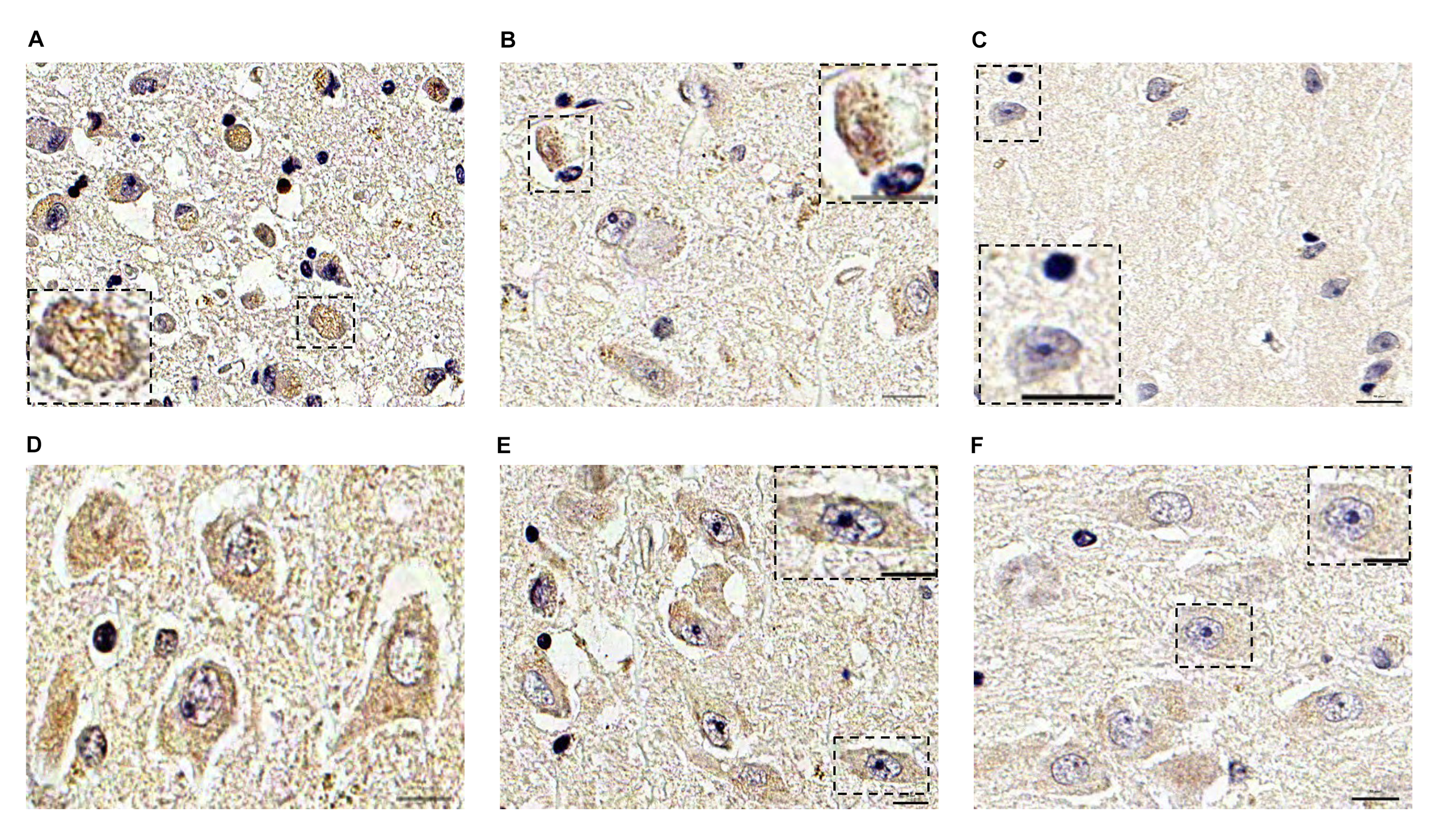

Supplement: Figure S9 — Hippocampus stained for Cdk4. Immunohistochemical immunoreactivity of Cdk4 in the nuclei (top) of hippocampal neurons from CP-AD (A), P-AD (B), and N (C) individuals. Immunohistochemical immunoreactivity of Cdk4 in the cytoplasm (bottom) of hippocampal neurons from CP-AD (D), P-AD (E), and N (F) individuals. Larger dashed boxes show magnifications of the smaller boxes. CP-AD, clinical-pathological Alzheimer’s disease; P-AD, pathological Alzheimer’s disease; N, normal aging. Scale bars = 50 pixels. (TIF) [file pone.0099897.s009.tif]

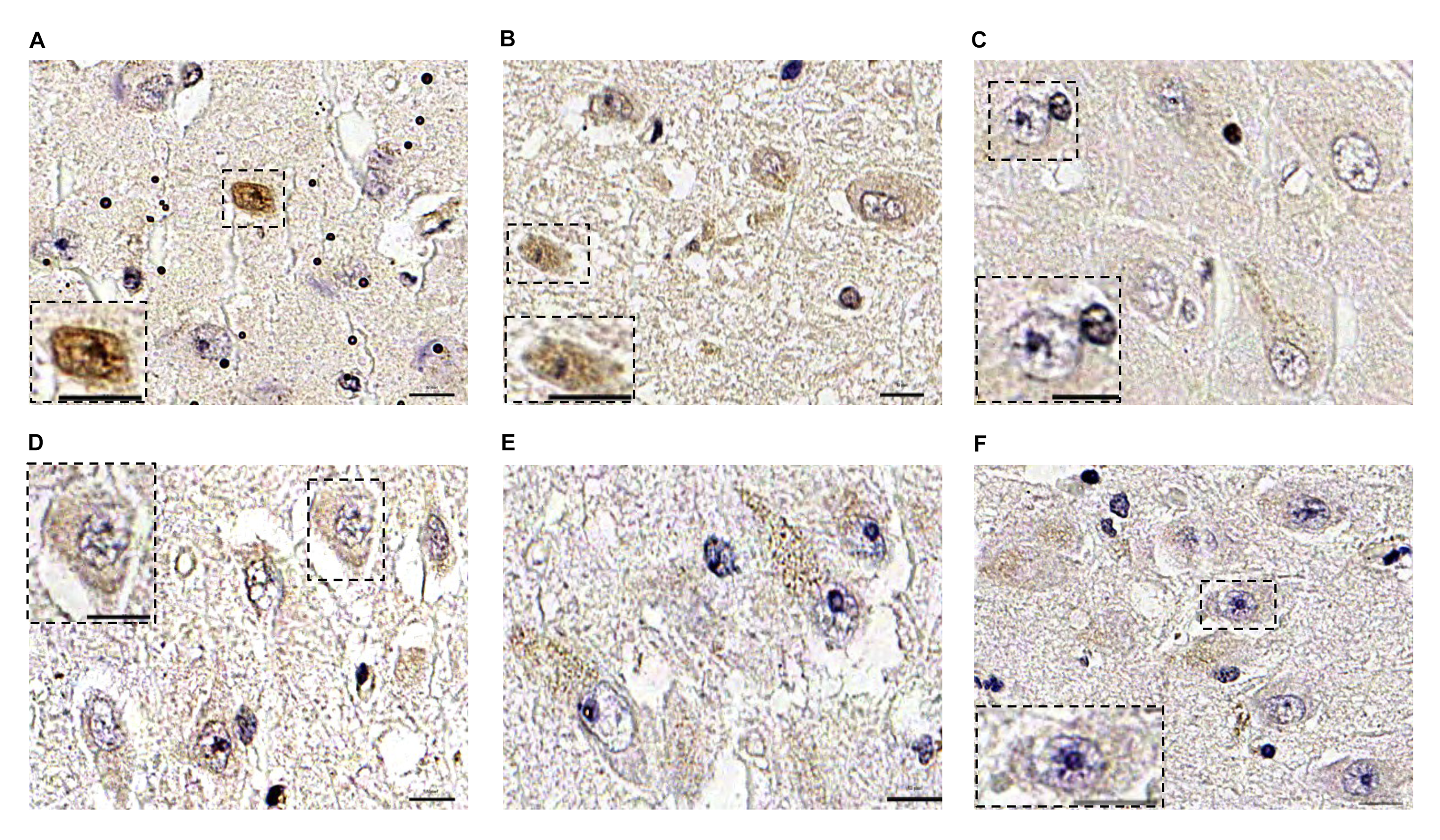

Supplement: Figure S10 — Hippocampus stained for Cyclin D. Immunohistochemical immunoreactivity of cyclin D in the nuclei (top) of hippocampal neurons from CP-AD (A), P-AD (B), and N (C) individuals. Immunohistochemical immunoreactivity of cyclin D in the cytoplasm (bottom) of hippocampal neurons from CP-AD (D), P-AD (E), and N (F) individuals. Larger dashed boxes show magnifications of the smaller boxes. CP-AD, clinical-pathological Alzheimer’s disease; P-AD, pathological Alzheimer’s disease; N, normal aging. Scale bars = 50 pixels. (TIF) [file pone.0099897.s010.tif]

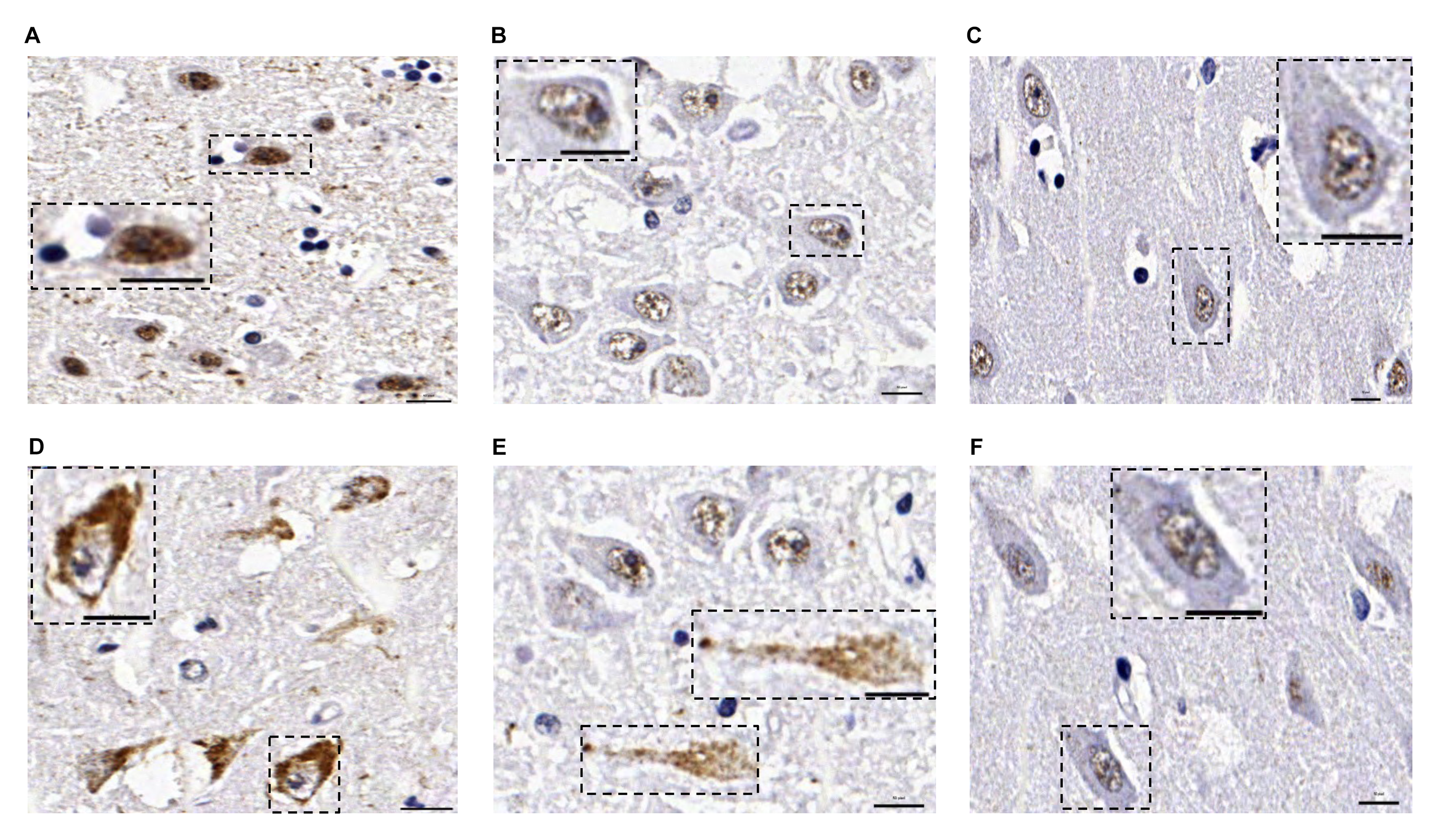

Supplement: Figure S11 — Hippocampus stained for phospho-Rb. Immunoreactivity of phospho-Rb in the nuclei (top) of hippocampal neurons from CP-AD (A), P-AD (B), and N (C) individuals. Immunoreactivity of phospho-Rb in the cytoplasm (bottom) of hippocampal neurons from CP-AD (D), P-AD (E), and N (F) individuals. Larger dashed boxes show magnifications of the smaller boxes. CP-AD, clinical-pathological Alzheimer’s disease; P-AD, pathological Alzheimer’s disease; N, normal aging. Scale bars = 50 pixels. (TIF) [file pone.0099897.s011.tif]

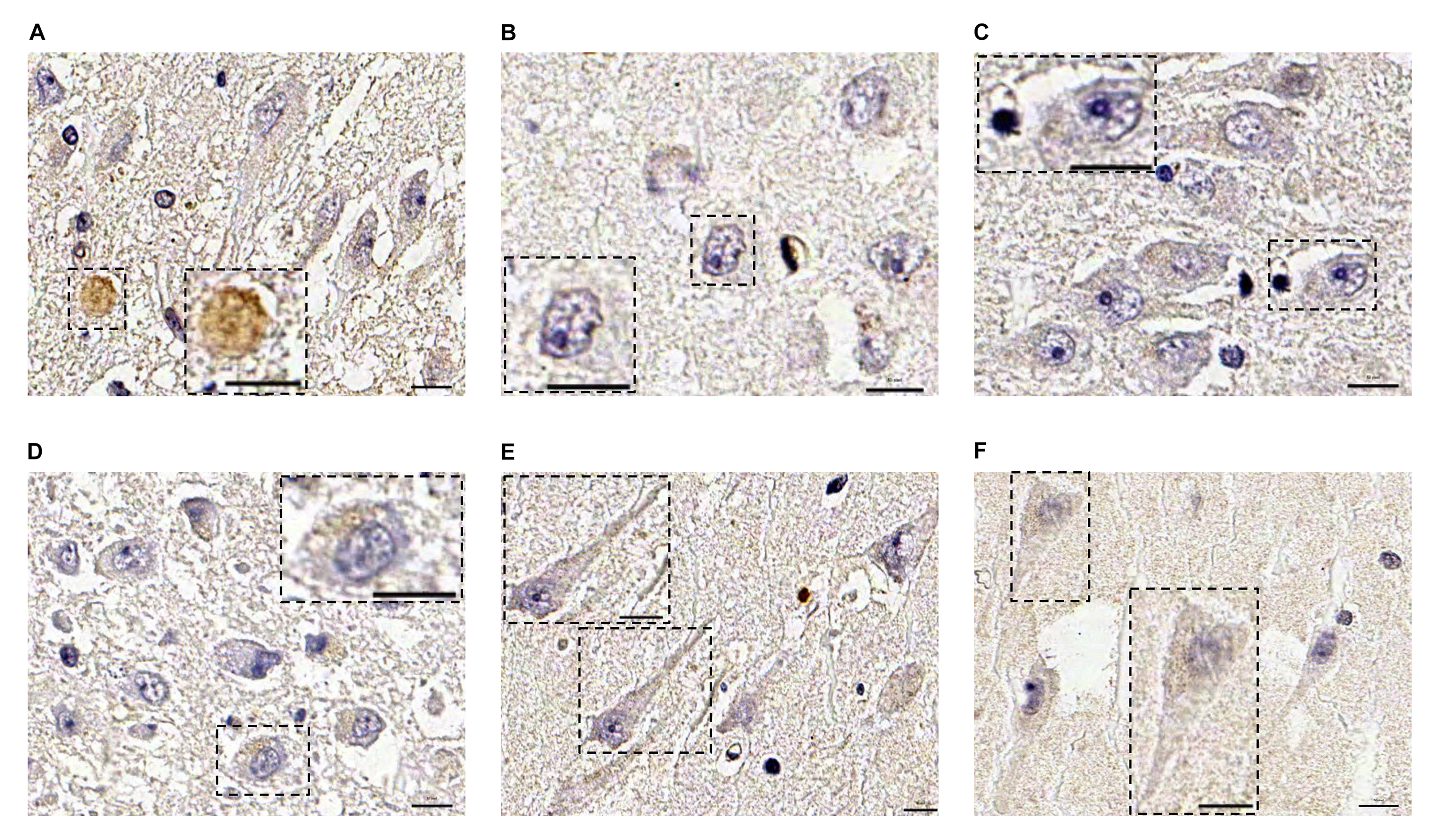

Supplement: Figure S12 — Hippocampus stained for E2F1. Immunoreactivity of E2F1 in the nuclei (top) of hippocampal neurons from CP-AD (A), P-AD (B), and N (C) individuals. Immunoreactivity of E2F1 in the cytoplasm (bottom) of hippocampal neurons from CP-AD (D), P-AD (E), and N (F) individuals. Larger dashed boxes show magnifications of the smaller boxes. CP-AD, clinical-pathological Alzheimer’s disease; P-AD, pathological Alzheimer’s disease; N, normal aging. Scale bars = 50 pixels. (TIF) [file pone.0099897.s012.tif]

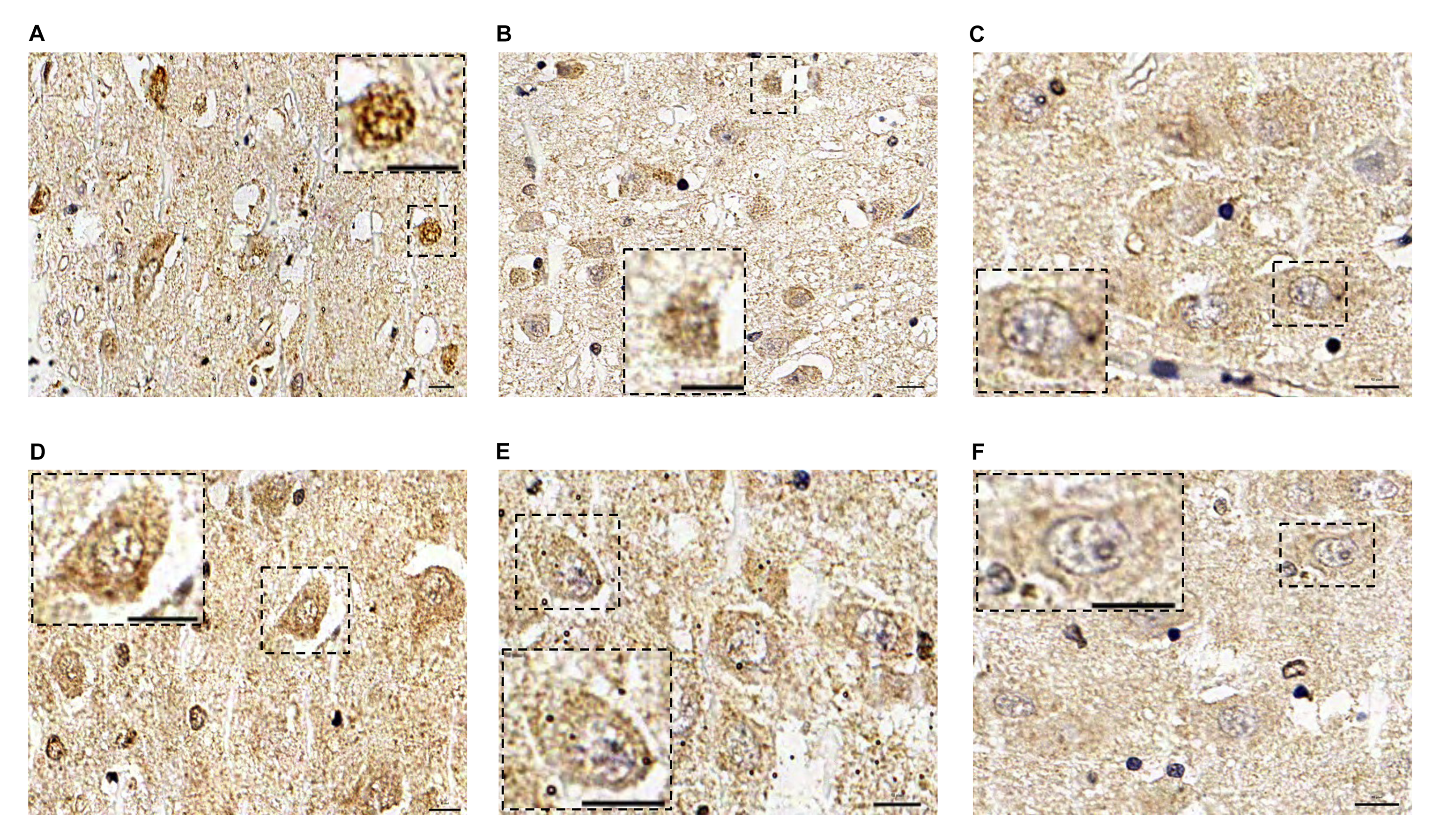

Supplement: Figure S13 — Hippocampus stained for Cdk1. Immunoreactivity of Cdk1 in the nuclei (top) of hippocampal neurons from CP-AD (A), P-AD (B), and N (C) individuals. Immunoreactivity of Cdk1 in the cytoplasm (bottom) of hippocampal neurons from CP-AD (D), P-AD (E), and N (F) individuals. Larger dashed boxes show magnifications of the smaller boxes. CP-AD, clinical-pathological Alzheimer’s disease; P-AD, pathological Alzheimer’s disease; N, normal aging. Scale bars = 50 pixels. (TIF) [file pone.0099897.s013.tif]

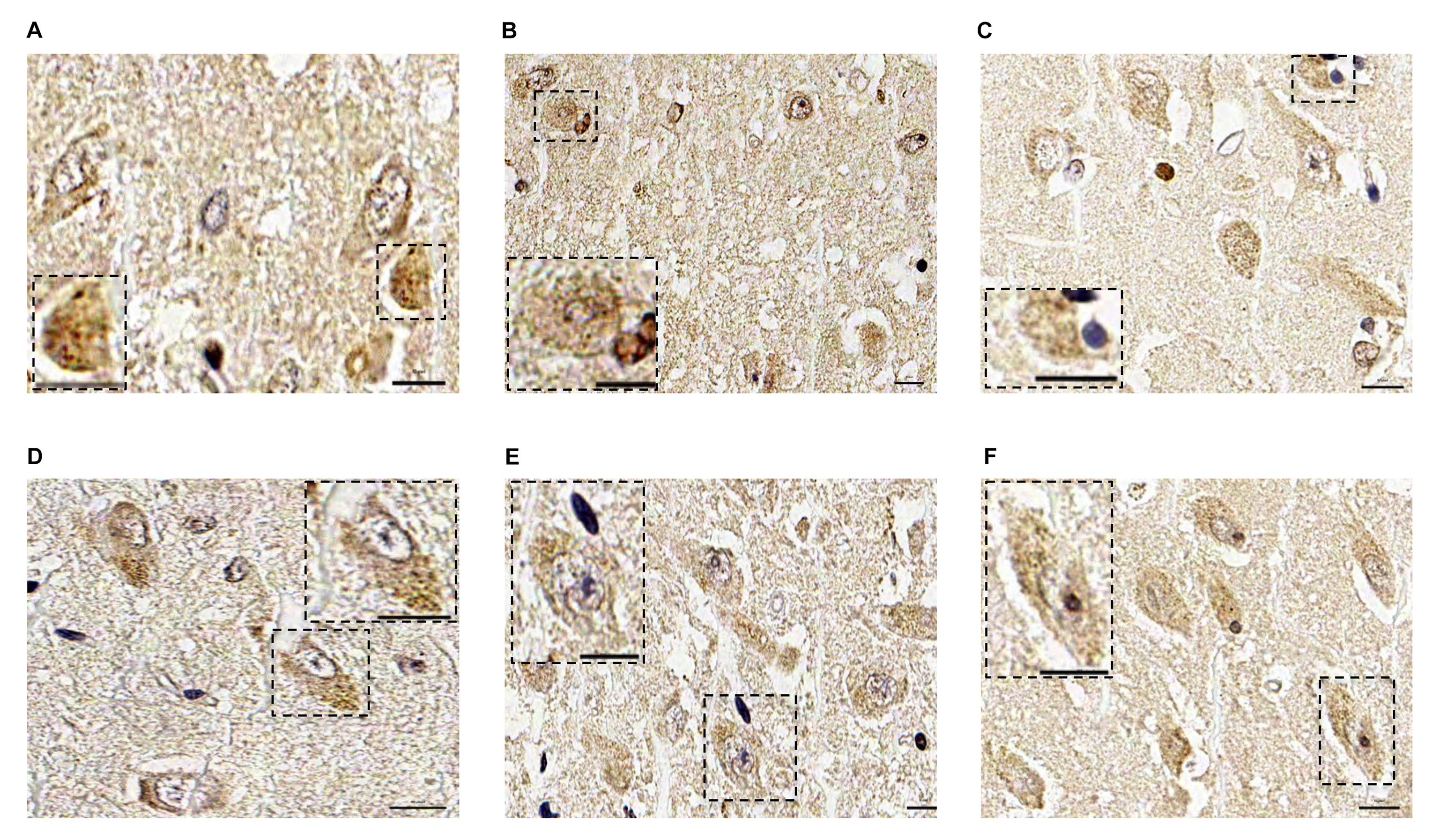

Supplement: Figure S14 — Hippocampus stained for cyclin B. Immunoreactivity of cyclin B in the nuclei (top) of hippocampal neurons from CP-AD (A), P-AD (B), and N (C) individuals. Immunoreactivity of cyclin B in the cytoplasm (bottom) of hippocampal neurons from CP-AD (D), P-AD (E), and N (F) individuals. Larger dashed boxes show magnifications of the smaller boxes. CP-AD, clinical-pathological Alzheimer’s disease; P-AD, pathological Alzheimer’s disease; N, normal aging. Scale bars = 50 pixels. (TIF) [file pone.0099897.s014.tif]

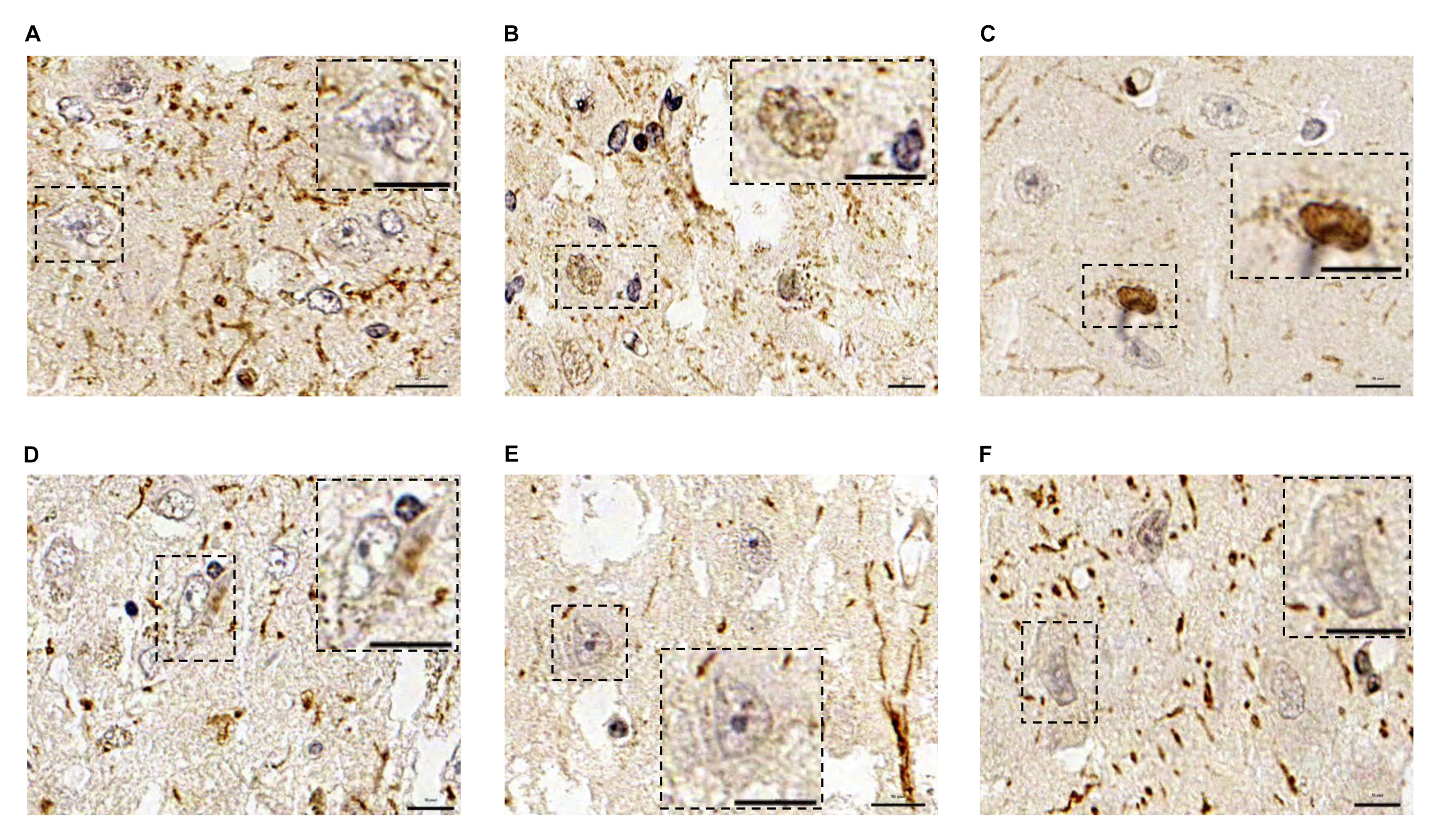

Supplement: Figure S15 — Hippocampus stained for p27. Immunoreactivity of p27 in the nuclei (top) of hippocampal neurons from CP-AD (A), P-AD (B), and N (C) individuals. Immunoreactivity of p27 in the cytoplasm (bottom) of hippocampal neurons from CP-AD (D), P-AD (E), and N (F) individuals. Larger dashed boxes show magnifications of the smaller boxes. CP-AD, clinical-pathological Alzheimer’s disease; P-AD, pathological Alzheimer’s disease; N, normal aging. Scale bars = 50 pixels. (TIF) [file pone.0099897.s015.tif]

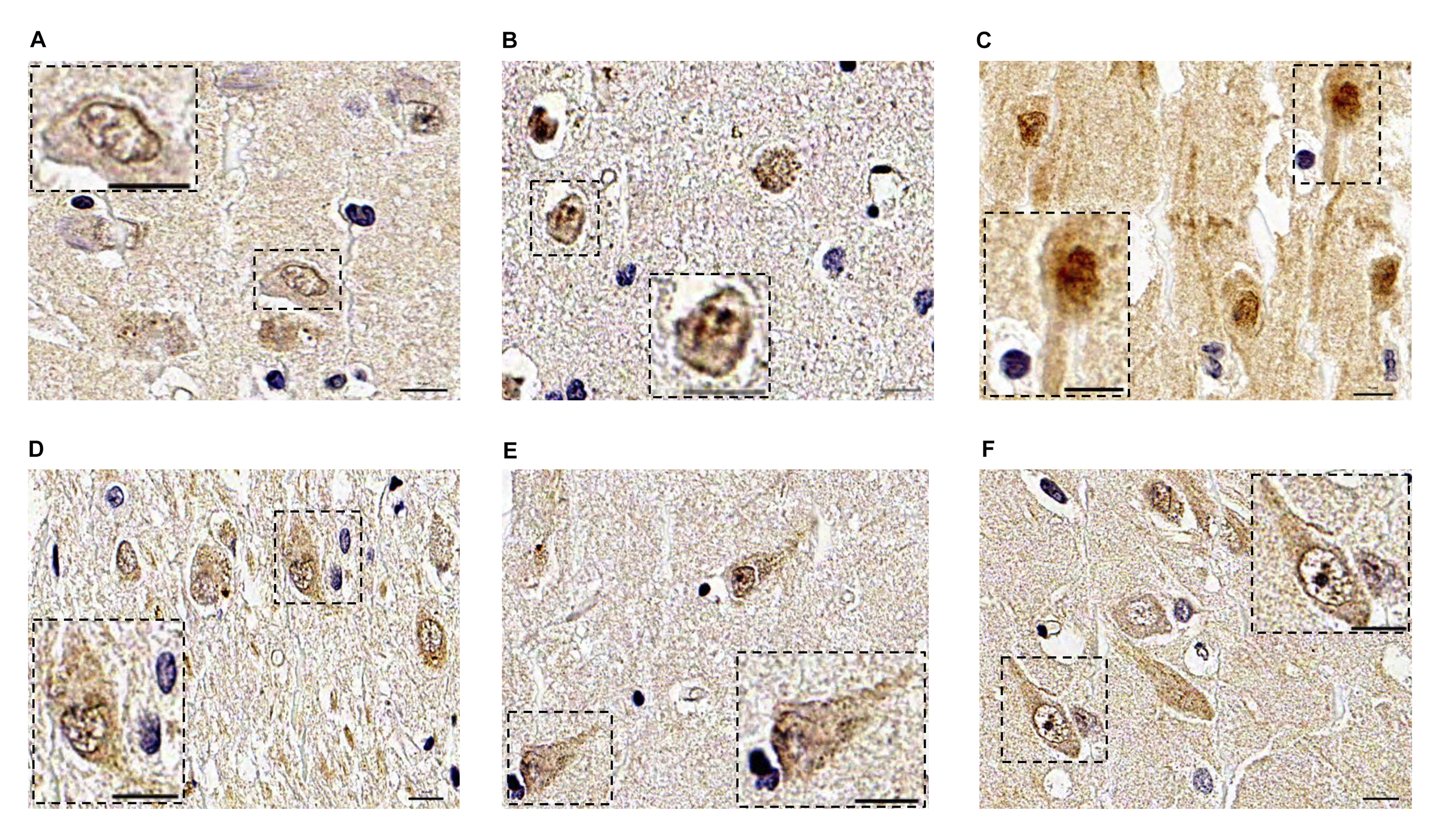

Supplement: Figure S16 — Hippocampus stained for Cdk5. Immunoreactivity of Cdk5 in the nuclei (top) of hippocampal neurons from CP-AD (A), P-AD (B), and N (C) individuals. Immunoreactivity of Cdk5 in the cytoplasm (bottom) of hippocampal neurons from CP-AD (D), P-AD (E), and N (F) individuals. Larger dashed boxes show magnifications of the smaller boxes. CP-AD, clinical-pathological Alzheimer’s disease; P-AD, pathological Alzheimer’s disease; N, normal aging. Scale bars = 50 pixels. (TIF) [file pone.0099897.s016.tif]
